# NMR Metabolomics and Chemometrics of Commercial Varieties of *Phaseolus vulgaris* L. Seeds from Italy and In Vitro Antioxidant and Antifungal Activity

**DOI:** 10.3390/plants13020227

**Published:** 2024-01-13

**Authors:** Vadym Samukha, Francesca Fantasma, Gilda D’Urso, Claudio Caprari, Vincenzo De Felice, Gabriella Saviano, Gianluigi Lauro, Agostino Casapullo, Maria Giovanna Chini, Giuseppe Bifulco, Maria Iorizzi

**Affiliations:** 1 Department of Biosciences and Territory, University of Molise, Contrada Fonte Lappone, 86090 Isernia, Italy; v.samukha@studenti.unimol.it (V.S.); fantasma@unimol.it (F.F.); claudio.caprari@unimol.it (C.C.); defelice@unimol.it (V.D.F.); saviano@unimol.it (G.S.); iorizzi@unimol.it (M.I.); 2 Department of Pharmacy, University of Salerno, Via Giovanni Paolo II 132, 84084 Salerno, Italy; gidurso@unisa.it (G.D.); glauro@unisa.it (G.L.); casapullo@unisa.it (A.C.)

**Keywords:** *Phaseolus vulgaris* L., food fingerprint, NMR metabolomics, nutraceutical profile, in vitro antioxidant and antifungal activity

## Abstract

The metabolite fingerprinting of four Italian commercial bean seed cultivars, i.e., *Phaseolus* Cannellino (PCANN), Controne (PCON), Vellutina (PVEL), and Occhio Nero (PON), were investigated by Nuclear Magnetic Resonance (NMR) spectroscopy and multivariate data analysis. The hydroalcoholic and organic extract analysis disclosed more than 32 metabolites from various classes, i.e., carbohydrates, amino acids, organic acids, nucleosides, alkaloids, and fatty acids. PVEL, PCON, and PCANN varieties displayed similar chemical profiles, albeit with somewhat different quantitative results. The PON metabolite composition was slightly different from the others; it lacked GABA and pipecolic acid, featured a higher percentage of malic acid than the other samples, and showed quantitative variations of several metabolites. The lipophilic extracts from all four cultivars demonstrated the presence of omega-3 and omega-6 unsaturated fatty acids. After the determination of the total phenolic, flavonoids, and condensed tannins content, in vitro antioxidant activity was then assessed using the DPPH scavenging activity, the ABTS scavenging assay, and ferric-reducing antioxidant power (FRAP). Compared to non-dark seeds (PCON, PCANN), brown seeds (PVEL, PON) featured a higher antioxidant capacity. Lastly, only PON extract showed in vitro antifungal activity against the sclerotia growth of *S. rolfsii*, by inhibiting halo growth by 75%.

## 1. Introduction

The common bean (*Phaseolus vulgaris* L.) has long been one of the most important traditional dietary food crops worldwide for both human consumption and animal feed, and it is the most produced and consumed legume in Africa, India, Latin America, and Mexico [1,2,3,4,5].

Beans (*Phaseolus* spp.) belong to the legume family (Fabaceae) and are considered the legume species with the highest distribution and consumption of the genus *Phaseolus*, which comprises about 150 known species of beans around the world [6,7]. Most species are endemic in several regions and represent more than 90% of the crops grown in the world [8].

As with other legumes, kidney bean seeds play an important role in the nutrition of low-income people, especially in developing countries, where they are often the most important dietary source of protein (20–25%), complex carbohydrates (50–60%), soluble-fibre starch, minerals, micronutrients, and vitamins [3,9,10].

Moreover, interesting biological activity has also been found in flavonol glycosides, anthocyanins, and condensed tannins (proanthocyanidins), which impart colour to the seed [11]. Several clinical studies have demonstrated the beneficial effect of bean consumption on the glycemic index with a protective role against type 2 diabetes because of their high polyphenol content, which possesses both antioxidant and anticarcinogenic properties [12]. Furthermore, bean consumption decreases the risk of ischemic heart and cardiovascular diseases, stomach and prostate cancer, weight control and obesity, stress attenuation, anxiety, and depression in elderly populations, and beans are endowed with antibacterial properties, among other roles [13,14,15,16,17].

The phytochemical composition of *Phaseolus vulgaris* L. seeds is the result of the evolution of the genetic diversity of the crops over the years [18]. Other important external factors are also involved in the qualitative and quantitative analysis of primary and secondary metabolites, such as the growing conditions, the microclimate of agricultural environments, seed characteristics (size, shape, and colour), ripening time, and storage conditions [19,20]. Bean consistency, taste, flavour, and health-related properties strictly depend on the seed’s chemical composition and on the balance between component levels.

The carbohydrate content is mainly represented by starch, constituting more than 50% of the seed weight, raw fibre, and less but significant amounts of mono-, di-, and oligosaccharides [21,22].

The absence of α-galactosidase in humans, which digest oligosaccharides, induces the anaerobic fermentation process of the oligosaccharides by microorganisms in the large intestine with the production of carbon dioxide, hydrogen, and methane, and can cause flatulence and diarrhoea [23,24,25].

The forms of consumption are varied. The dry grains (ripe) of *P. vulgaris* are generally boiled for further consumption. Still, the fresh leaves and young pods can also be used in traditional dishes, as edible vegetables [6] or as an additive to prepare soups. The green pods are consumed fresh, frozen, as part of mixes, or as canned vegetables [26], or as a substitute for gluten-free wheat flour [27].

Legumes are a rich source of bioactive phenolic or polyphenolic compounds, tocopherols, and triterpenic acids [28]. In humans, besides contributing to the smell, taste, and colour of food, polyphenols play a significant role in many physiological and metabolic processes [29]. These beneficial effects may be attributed to their capacity to inhibit the activity of enzymes correlated to the production of reactive oxygen species and superoxide anions [30], and they also act as chelators of metal ions capable of catalysing oxidative reactions [31]. Polyphenols exert a modulatory effect on different components of the cells, having an important role in cellular functions such as growth, proliferation, and apoptosis [32].

As a result, the bioactive phenolic compounds in grain legumes make them suitable candidates for creating new functional foods [33]. The phytochemical investigation of several *Phaseolus* species revealed a considerable number of flavonoids, phenolic acids, anthocyanins, and condensed tannins (proanthocyanidins), which are the main phenolic compounds identified and characterised in beans [34]. However, phenolic compounds can vary between bean seeds, with dark seeds having the highest concentration compared to the light-coloured varieties [35]. Anthocyanins have been detected in more pigmented beans [36,37,38], and the anthocyanins from black bean coats have recently attracted significant interest in the food industry as natural colorants and, as alternative sources to synthetic additives [39]. Condensed tannins (CTs), also called proanthocyanidins, are complex polymers of flavan-3-ol units, the number of which reflects the degree of polymerisation [40]. CTs are also responsible for colour, and recently, it has been suggested that any potential health benefits attributed to these compounds may be affected by the degree of polymerisation [41].

Many studies on bean cultivars have investigated kidney bean phenolic profile and antioxidant capacity by analysing traditional cultivars. Reversed-phase liquid chromatography (HPLC/UPLC) coupled with either diode array detection (DAD) [36,42] or high-resolution mass spectrometry (HRMS) [43,44] are the techniques of choice for the analysis of individual phenols. Several methods have been developed and applied to measure the antioxidant activity in legumes: those based on the scavenging activity toward a stable free radical (2,2-diphenyl-1-picrylhydrazyl (DPPH) and 2,2′-azino-bis(3-ethylbenzothiazoline-6-sulfonic acid) (ABTS), or on the reduction of metal ions, ferric ion-reducing antioxidant potential (FRAP), cupric ion-reducing antioxidant capacity (CUPRAC), Folin-Ciocalteu (FC) assay, or competitive methods (oxygen radical absorbance capacity (ORAC), total reactive antioxidant potential (TRAP)) [45,46]. Several antioxidant properties have been reported with respect to *P. vulgaris*, highlighting the health-promoting properties of whole bean consumption [47,48,49]. Although legumes are protein-rich foods, they are lacking in sulphur-containing amino acids methionine and cysteine [3] and, to some extent, tryptophan [50]. On the other hand, cereals contain sulphur amino acids but are limited in the essential amino acid lysine; hence, a combination of legumes and cereals would improve the protein and nutrient density of the subsequent food products. The lipid fraction of dry common beans is the least representative, found to be 2.20–5.03%, and it is composed of an acyl-glycerides mixture of mono- and polyunsaturated fatty acids with the most concentrated linoleic and linolenic fatty acids [6,51]. Despite their positive characteristics, beans also contain some antinutritional components, e.g., tannins, lectins, phytic acid, and hemagglutinins, that limit their nutritional value [52]. The consumption of raw or inadequately cooked beans is known to cause poisoning, characterised by nausea, vomiting, diarrhoea, severe acute gastroenteritis, and intestinal malabsorption [53]. This toxicity has been attributed to the presence of active lectins, one of the major classes of common bean storage proteins [54]. Lectins induce the growth of the pancreas, producing ulceration and necrosis in the intestinal epithelium of rats. For these reasons, without prior heat treatment, the presence of lectins severely limits the use of common bean flour and cooking conditions and only sometimes results in their complete inactivation, so this is a limitation to preparing baked products. In addition, common bean seeds contain high amounts of cation chelator phytic acid, which reduces the bioavailability of essential minerals such as iron, zinc, potassium, calcium, and magnesium that easily precipitate as phytate salts at physiological pH [55]. Tannins can also reduce food consumption and the bioavailability of minerals [7]. Condensed tannins have been generally reported as antinutritional factors; however, CTs are also considered to be powerful radical scavengers.

Due to the economic, cultural, and nutritional importance of *Phaseolus* cultivars, this study aimed to identify and quantify the significant metabolites in four commercial bean seeds, i.e., *Phaseolus* Cannellino (PCANN), *Phaseolus* Controne (PCON), *Phaseolus* Vellutina (PVEL), and *Phaseolus* Occhio Nero (PON), having different colours and shapes, using the Nuclear Magnetic Resonance (NMR) methodology to determine the differences (metabolomics fingerprinting). Knowledge of *Phaseolus* variety profiles can be important for introducing local products concerning their nutritional content to national markets.

The ultimate aim of this study was to investigate and identify the range of organic compounds in the hydroalcoholic and organic extracts of four different commercial dry bean seed genotypes by non-targeted and targeted chemometric analysis using ^1^H NMR-based metabolomics in combination with multivariate data analysis (MVDA). Furthermore, to complete the characterisation, the total phenolic content, total flavonoids, and condensed tannins content were determined in ethanolic extracts, followed by in vitro antioxidant capability using the DPPH, ABTS, and FRAP methods, as well as their antifungal activity.

## 2. Results and Discussion

### 2.1. Metabolomic Analysis: NMR Assignment of the Aqueous Extracts

**Figure 1:**
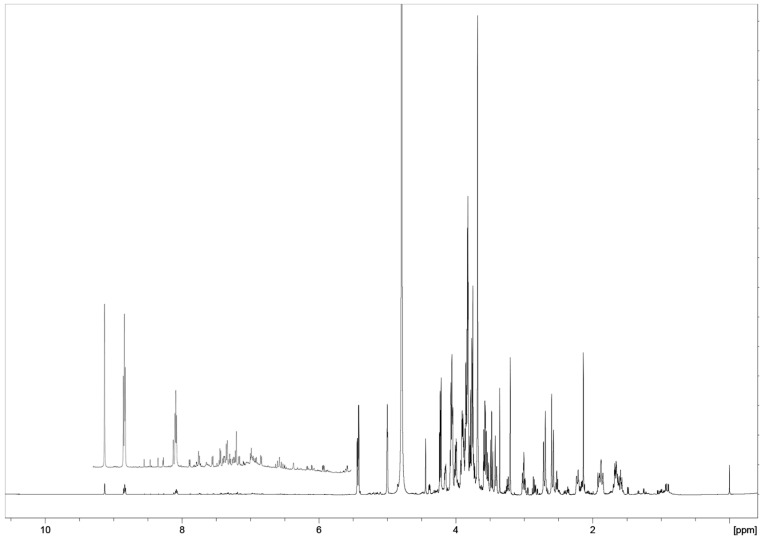
Characteristic ^1^H NMR spectrum obtained at 600 MHz (D_2_O) of aqueous extracts from seeds of *Phaseolus* Vellutina (PVEL) and its expanded region of the ^1^H NMR spectrum from 5.5 to 9.3 ppm.

A metabolomic approach using NMR spectroscopy coupled with multivariate data analysis provided a detailed metabolite profile of the hydroalcoholic and organic extracts of the tested commercial *Phaseolus* cultivars (Figure 1): *Phaseolus* Cannellino (PCANN), *Phaseolus* Controne (PCON), *Phaseolus* Vellutina (PVEL), and *Phaseolus* Occhio Nero (PON). The ^1^H (Figure 1 and Figures S1–S3) and ^13^C NMR assignments were also supported by 2D-NMR experiments (COSY, HSQC, HMBC, see Figures S4–S6 in SI), standard samples available in the laboratory, literature data, and comparison of NMR data (1D and 2D) with the data in the Human Metabolome Data Base (HMDB; https://hmdb.ca/, accessed on 31 October 2023), and by using Chenomx NMR-Suite v 9.0 (Chenomx Inc., Edmonton, AB, Canada). In the ^1^H NMR spectra of aqueous extracts (Figure 1 and Figures S1–S3), most high-intensity signals belong to primary metabolites, i.e., sugars, organic acids, and amino acids. In contrast, much lower intensity signals were observed for the aromatic amino acids and other aromatic components. ^1^H NMR data reported in Table 1 were extrapolated from the *Phaseolus* Vellutina (PVEL) sample (Figure 1).

**Figure 2:**
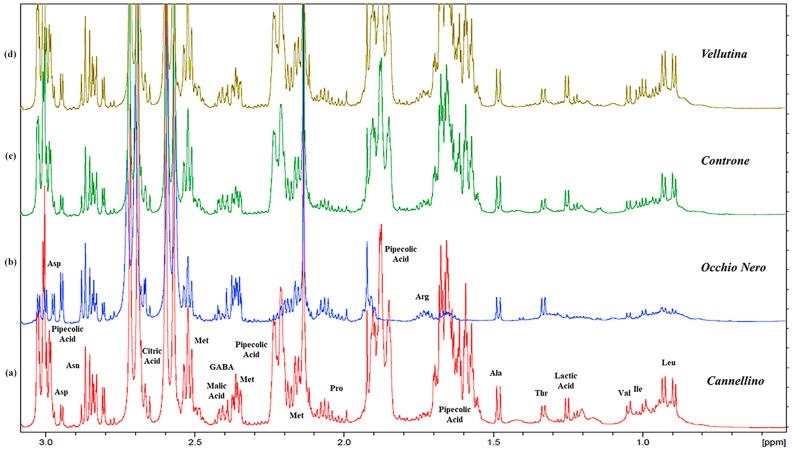
Expanded region (from 0.00 to 3.00 ppm) of ^1^H NMR spectra obtained at 600 MHz (D_2_O) of aqueous extracts of each commercial bean variety selected for the analysis: (**a**) *Phaseolus* Cannellino (PCANN) is reported in red; (**b**) *Phaseolus* Occhio Nero (PCON) is reported in blue; (**c**) *Phaseolus* Controne (PCON) is reported in green; (**d**) *Phaseolus* Vellutina (PVEL) is reported in dark yellow.

**Figure 3:**
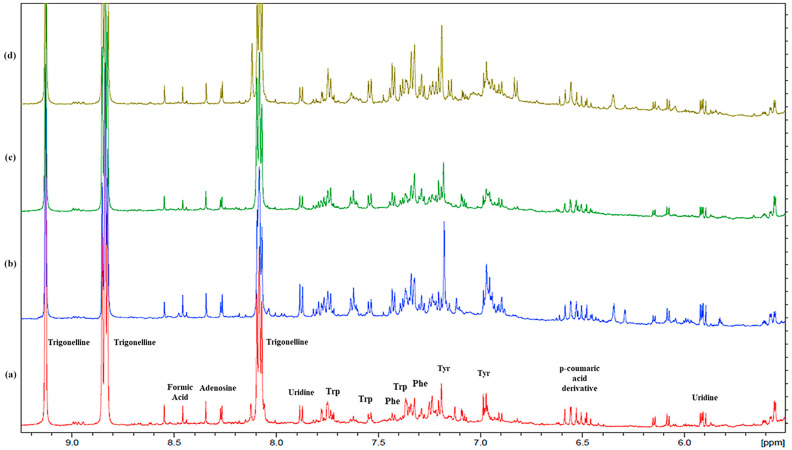
Expanded region (from 6.00 to 9.00 ppm) of ^1^H NMR spectra obtained at 600 MHz (D_2_O) of aqueous extracts of each commercial bean variety selected for the analysis: (**a**) *Phaseolus* Cannellino (PCANN) is reported in red; (**b**) *Phaseolus* Occhio Nero (PCON) is reported in blue; (**c**) *Phaseolus* Controne (PCON) is reported in green; (**d**) *Phaseolus* Vellutina (PVEL) is reported in dark yellow.

**Figure 4:**
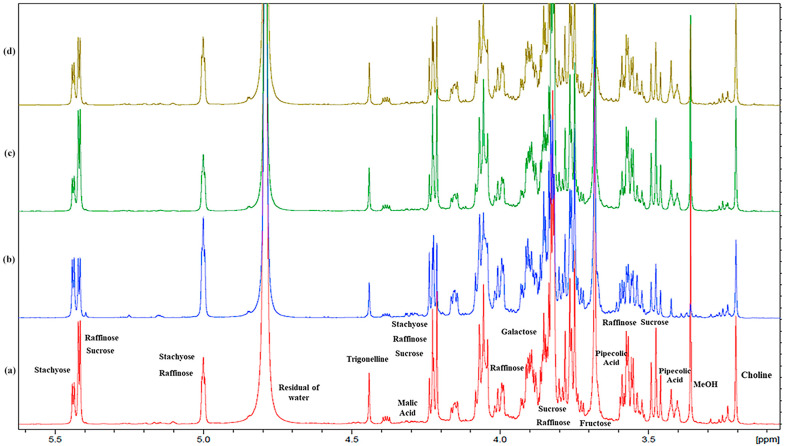
Expanded region (from 3.00 to 6.00 ppm) of ^1^H NMR spectra obtained at 600 MHz (D_2_O) of aqueous extracts of each commercial bean variety selected for the analysis: (**a**) *Phaseolus* Cannellino (PCANN) is reported in red; (**b**) *Phaseolus* Occhio Nero (PCON) is reported in blue; (**c**) *Phaseolus* Controne (PCON) is reported in green; (**d**) *Phaseolus* Vellutina (PVEL) is reported in dark yellow.

The results revealed qualitative and quantitative differences between the *Phaseolus* samples (*vide infra*). We assigned the signals to 29 compounds in the polar extracts, including amino acids, organic acids, carbohydrates, nucleosides, and phenolics (Table 1). The ^1^H NMR spectra of the seeds of four Italian commercial cultivars were very complex (Figure 1 and Figures S1–S3) and showed a similar chemical profiling (Figure 2, Figure 3, Figure 4 and Figures S1–S3), but differences in the intensity and relative abundance of metabolites in the sample PVEL, PCON, and PCANN. The metabolite composition was slightly different in the PON sample, where some components were missing (*vide infra*). Many signals in the ^1^H NMR spectra of all samples were partially overlapped. 

#### 2.1.1. Free Amino Acids

Several free amino acids were identified by their diagnostic chemical shift values (Figure 1, Figure 2, and Figures S1–S3). In the aliphatic region, the branched amino acids valine (Val) and leucine (Leu) were evidenced by their methyl signals at δ 0.99 and 1.05 (Val), while leucine showed signals at δ 0.93 and 0.90 (Table 1). In addition, threonine (Thr) (1.33, d) and alanine (Ala) (1.48, d) were detected. Further diagnostic resonances included those found for the methionine (Met) with the -S-CH_3_ signal at δ 2.14 (s) and the β-CH_2_ signal at δ 2.38 partially overlapped, asparagine (Asn) with a dd assigned at β-CH_2_ at δ 2.86 (*J *= 7.3, 16.8 Hz) and aspartate (Asp) with a signal of β-CH_2_ at δ 3.02 (dd, *J *= 6.8, 17.3 Hz) and 2.95 ppm (from ^1^H NMR of sample PON). Then, glutamic acid (Glu) was detected based on α-CH_2_ and γ-CH_2_ resonating at 2.04/2.14 (m) and 2.36 (m), respectively. The presence of GABA was confirmed by its typical triplet assigned to the α-CH_2_ protons (2.30 t, *J *= 7.3 Hz) and γ-CH_2_ (2.99, t), the latter partially overlapped with other signals, and by the aid of 2D-NMR data.

Gamma-Aminobutyrate (GABA) is a ubiquitous, non-proteinogenic amino acid that, in plants, is associated with the metabolism of carbohydrates and amino acids, and acts as a defence against various abiotic and biotic stresses [56]. It is widely present in all living organisms, such as microorganisms, animals, insects, worms, and plants [57]. In humans, GABA acts as a depressive neurotransmitter in the central nervous system, and it can also regulate blood pressure and relieve pain and anxiety [58]. It is known that during seed germination, the accumulation of many bioactive compounds occurs, including polyphenols, vitamins, and GABA. In bean seeds, it has been reported that GABA is generally found at a low level [59]; however, its level rapidly increases during the germination phase.

In the four samples, we detected low concentrations of GABA in PCANN, PVEL, and PCON (Figure 2), as reported in the literature. In contrast, GABA was missing in the PON sample. This behaviour could be related to the genetic characteristic of PON, and GABA is probably in undetectable concentrations compared to the other samples.

In the low field region (Figure 3), the aromatic amino acid signals of phenylalanine (Phe), tyrosine (Tyr), and tryptophan (Trp) resonated in a crowded part of the spectra (6.5–8.0 ppm) partially overlapped with other aromatic compounds signals such as polyphenols that could not be identified because of their very low concentration. These results are in line with previous NMR metabolomic analyses that reported leucine and methionine as the main free amino acids in beans [60,61].

Expansion of the aromatic region between 6.4 and 6.6 ppm (Figure 3) displays a series of doublets with a coupling constant (*J*) of 16.0/16.2 Hz. Based on the chemical shifts and (*J*) these signals can be attributed to α,β-unsaturated systems with *trans* configuration, typical for compounds derived from hydroxycinnamic acids widely distributed in the plant kingdom [62].

#### 2.1.2. Carbohydrates

Carbohydrates were the main compounds evidenced in the aqueous extract, as expected, since dry beans are a good source of soluble reducing sugars [10] and contain a high proportion of non-digestible carbohydrates. The carbohydrate region (Figure 4, 5.5–3.0 ppm) was rather complex; the main carbohydrates present in beans are the raffinose-family of oligosaccharides (RFOs) and sucrose [63]. RFOs represent a group of soluble but non-reducing and non-structural sugars characterised by the presence of α-(1 → 6) glycosidic linkages. Raffinose is the first member of this family, followed by stachyose and verbascose [64]. Sucrose, raffinose, and stachyose were identified through their diagnostic ^1^H NMR signals and 2D experiments. All accessions included sucrose/raffinose and stachyose in high concentrations, evidenced by their anomeric proton signals at 5.42 ppm (d, *J* = 4 Hz) and 5.44 ppm (d, *J* = 4 Hz) and 5.00 (brs), respectively. Sucrose is a disaccharide with glucose and fructose units, while raffinose and stachyose are mono- and di-galactosyl derivatives of sucrose. Their structures were confirmed by an inspection of the cross peak in the COSY and HSQC spectrum (Figures S4 and S5) and by comparison with the NMR data of Watson et al. [65].

#### 2.1.3. Organic Acids

Several organic acids were detected in the aqueous extract (Table 1). The presence of lactic acid was confirmed by its methyl group resonating at δ 1.25 (d, *J *= 7 Hz), while malic acid was evidenced by the signal at 4.30 (dd, partially overlapped). The characteristic signals of citric acid with the methylene hydrogens (AB spin system) were clearly visible at 2.58 and 2.70 ppm (*J *= 16.0 Hz), which correlated in the HSQC experiment with the corresponding carbon atom at 44.5 ppm (Figure S5). In the ^1^H-^1^H COSY experiment (Figure S4), the diagnostic chemical shifts of the spin systems, the coupling constants, and the correlations in the HSQC (Figure S5) experiment suggested the presence of pipecolic acid (Pip). The diagnostic signals were -CH_2_-6 at 3.41 (dd) and 3.00 (td, 12.5, 3.2), methine -CH-2 at 3.59 (dd), which correlated with the corresponding carbon signal at 43.5 ppm and 59.0 ppm. Greater complexity was observed for signals from -CH_2_-3 to -CH_2_-5 (1.67–2.22 ppm), which were followed by a cross peak in the COSY experiments (Figure S4). Pipecolic acid was found in PCANN, PVEL, and PCON, while it was missing in the PON sample (Figure 2). Thus, NMR experiments allowed us to detect arginine and aspartate signals that were partially masked by non-protein amino acid pipecolic acid (Pip) signals in the spectra of the other cultivars (Table 1).

In line with recent studies, high levels of organic acid provide carbon building blocks for the defensive compounds production in plant tissues and probably the role of phytoalexins at the sprout stage critical in plant life [66]. Moreover, organic acids are essential food components responsible for organoleptic characteristics [67]. Among organic acids, the non-protein amino acid pipecolic acid (Pip) is a lysine catabolite involved in plant systemic acquired resistance (SAR) and acts as a chemopreventive marker in beans [68]. It has been reported that Pip biosynthesis is strictly dependent on a functional ALD1 gene that encodes an aminotransferase with an in vitro substrate preference for Lys. Thus, mutations in the ALD1 gene prevent Pip accumulation and SAR formation [69]. Recently, it has also been recognised as an important regulator of immunity in humans [70], and it has been shown that Pip can be considered one of the metabolic markers of dry bean consumption, being the most abundant non-protein nitrogen fraction (6788 mg/kg dry beans) and a diet rich in beans has been shown to have chemopreventive effects on the risk of colorectal neoplasia in parallel human and mouse studies [71].

#### 2.1.4. Other Components

The -N^+^(CH_3_)_3_ group of choline was detected by the characteristic singlet at 3.21 ppm (Figure 4), which correlated with the methyl carbon at 53.6 ppm in the HSQC spectrum (Figure S5).

The alkaloid trigonelline (Figure 3 and Figure S7) was identified through multiple peaks at 9.13 (H-2, s), 8.83 (H-4 and H-6, m), 8.09 (H-5, m) ppm, and N-CH_3_ characteristic signals, and confirmed by the ^13^C NMR resonances at 145.7, 145.5, 127.7, and 48.2 ppm, assigned via the HSQC experiment (Table 1, Figures S4 and S5). Trigonelline, a vitamin B6 derivative, is widely distributed in dry legume seeds [72] and acts as a storage form in plants that turns into NAD^+^ during germination [73]. Its biological activities have long been evaluated, especially concerning diabetes and central nervous system diseases. Several studies have reported that trigonelline showed beneficial effects against diabetes β-cell regeneration, insulin secretion, and the activities of enzymes related to glucose metabolism. It has been evaluated against neurodegenerative diseases for its neuroprotective activity, and it also possesses antimigraine, sedative, memory-enhancing, and hypolipidemic activities. Moreover, it may reduce auditory neuropathy and platelet aggregation [74].

### 2.2. NMR Assignment of the Organic Extracts

**Figure 5:**
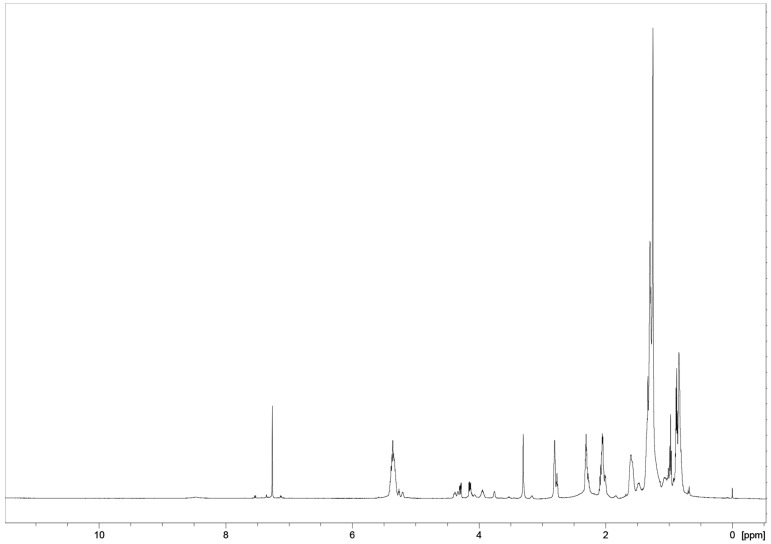
Spectrum in CDCl_3_ of apolar extract of *Phaseolus* Vellutina (PVEL) bean at 600 MHz.

Fatty acids (FAs) are the major lipid building blocks of complex lipids, such as glycerolipids, i.e., monoacylglycerols (MAGs), diacylglycerols (DAGs), and triacylglycerols (TAGs, Figure 5 and Figures S8–S13). These neutral lipids have a glycerol backbone with FA chains attached to the glycerol group, most commonly with an ester bond.

^1^H NMR spectra in CDCl_3_ were also acquired for the organic extracts of all samples (Figure 5 and Figures S8–S13).

According to the previously published data [6,51], the apolar fraction of *Phaseolus* samples was mainly composed of monounsaturated fatty acids (MUFA) and polyunsaturated fatty acids (PUFA). Their diagnostic chemical shifts are shown in Table 1.

Major signals (Table 1) were detected for the protons of the triacylglycerols (TAG) at 4.15 (dd) and 4.29 (dd) ppm (*sn*-1 and *sn*-3 H_2_) and 5.27 ppm (m, *sn*-2) and correlated with the corresponding carbon atoms at 61.8 and 68.1 ppm in the HSQC experiment (Figure S12). Additionally observed were: olefinic proton signals of unsaturated fatty acids (-CH=CH-) at ~5.37 ppm, the protons of α-CH_2_ (-CH_2_COO^−^) at ~2.33 ppm, β-CH_2_ (-CH_2_-CH_2_COO^−^) at 1.61 ppm, and -(CH_2_)_n_- at 1.25 and 1.30 ppm. The allylic protons were identified at ~2.06 ppm and showed a correlation with carbons at ~27.1 ppm in the HSQC (Figure S12). Very relevant were the signals at 2.76 and 2.80 ppm that could be attributed to bis-allylic protons of the -(CH_2_)- groups located between pairs of double bonds and thus provided a measure of the number of poly-unsaturated ω-3 and ω-6 fatty acids (PUFA) present in the samples [51]. Terminal methyl groups were identified at 0.89 ppm (t), indicative of α-linoleic acid, and 0.97 ppm (t), suggesting the presence of α-linolenic acid (18:3 ω-3), the methyl group of which was shifted to the low field due to the double bond close to the terminal –CH_3_ group. As reported by Tsiafoulis et al. [75], the methyl group at 0.89 ppm of α-linolenic acid correlated with the bis-allylic proton signals at 2.77 ppm, while the methyl signal at 0.97 ppm of α-linoleic acid (18:2) had the corresponding bis-allylic proton signals at 2.81 ppm. The above NMR chemical shifts data agreed with the Livestock Metabolome Database (LMDB), which confirmed the assignment of linoleic and α-linolenic acids.

Furthermore, diacylglycerides (DAG) signals (*sn*-1,2/2,3) were detected as a multiplet at 3.74 ppm (OH-CH_2_-CH), while the signal for *sn*-1,2 DAG resonates as dd at 4.36 ppm (1′*b* -CH_2_-O-CO-).

### 2.3. Quantitative Analysis

Quantitative NMR (qNMR) is a powerful tool that provides simultaneous access to both qualitative (chemical structure) and quantitative information. Most quantitative NMR assay methods are based on using an internal standard for particular analysis and improvement of the results.

For quantification, spectra of hydrophilic extracts were imported to NMRProcFlow software (version 1.4.20) [76] (https://nmrprocflow.org/, accessed on 15 November 2023), and NMR signals unique to each metabolite and sufficiently separated from neighbouring signals were selected and quantified.

**Figure 6:**
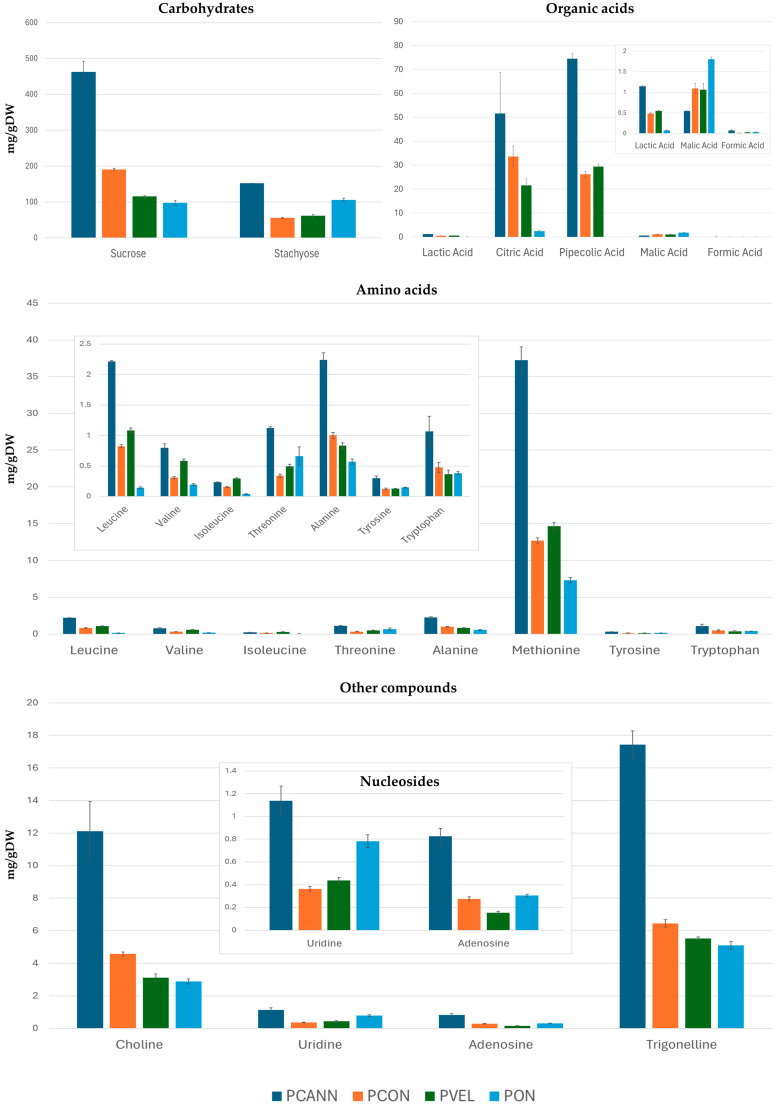
Abundances of metabolites identified in polar extracts of *Phaseolus* Cannellino (PCANN), *Phaseolus* Controne (PCON), *Phaseolus* Vellutina (PVEL), and *Phaseolus* Occhio Nero (PON).

qNMR carried out the relative quantification of selected metabolites of polar extracts, and the comparison of their abundances allowed the evaluation of their quantitative differences in the analysed commercial bean varieties, dividing the compounds into classes: amino acids, carbohydrates, organic acids, and other compounds, including nucleosides (Figure 6). Only compounds that showed a high variation in concentration and did not show overlapping resonances were quantified.

Stachyose and sucrose were the main carbohydrates detected in all samples. In PCANN, PCON, and PVEL, sucrose was found to exceed that of stachyose in concentration. Only in the PON sample was the concentration of both carbohydrates almost identical. Among the organic acids, pipecolic acid was present in the highest amount in PCANN; its concentration decreased in PVEL and PCON, and then disappeared in the PON sample. The lack of pipecolic acid in the PON sample allowed us to quantify the content of arginine and aspartate with a higher percentage of arginine compared to aspartate. In Table 1, the signals chosen for the quantification of the corresponding compounds are indicated with (#). Citric, malic, and lactic acids were detected and quantified in all samples and differences were observed. Citric acid was abundant in PCANN and decreased in PCON and PVEL, while it was in low concentrations in PON. Compared to citric acid, lactic and malic acid were in much lower concentrations; however, in the PON sample, the amount of malic acid was higher than in the other varieties. Among the amino acids, methionine dominated in all the samples analysed, with the highest quantification in PCANN, decreasing in PVEL, PCON, and PON. Concentrations gradually decreased for Ala, Leu, Thr, Trp, Val, Ile, and Tyr in the four samples. Leucine and alanine in PCANN were almost identical.

Trigonelline and choline were detected in all *Phaseolus* accessions. PCANN had the largest quantities of both components, and their concentrations progressively decreased, moving to PCON, PVEL, and PON. A similar behaviour was observed for nucleosides.

In summary, the PCANN, PCON, and PVEL varieties are quite similar in their composition of carbohydrates, amino acids, and organic acids but differ in their concentrations. Differences emerge for the PON variety in which pipecolic acid and GABA disappear, the concentration of sucrose and stachyose is almost similar, and malic acid dominates the other organic acids.

### 2.4. Chemometric Analysis

**Figure 7:**
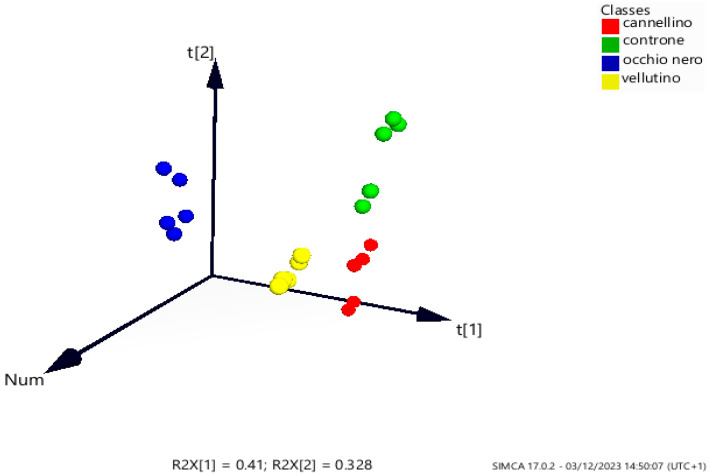
3D representation of Principal Component Analysis (PCA) of polar extracts of bean varieties. PCON “*Phaseolus* Controne” (green), PVEL “*Phaseolus* Vellutina” (yellow), and PCANN “*Phaseolus* Cannellino” (red) separated on the right part of the plot, while the PON “*Phaseolus* Occhio Nero” variety (blue) is separated in the score plot.

Untargeted and targeted multivariate data analyses were performed to establish metabolic differences in the hydroalcoholic extracts among the selected commercial bean varieties. For the untargeted approach, the matrix of integrated ^1^H NMR spectral data, both with unknown and known metabolites, was imported into Simca software version 17 for the construction of Principal Component Analysis (PCA). Therefore, five replicates (n = 20) were carried out for each variety. The score scatter plot of PCA (Figure 7) shows four different clusters with PCON “*Phaseolus* Controne” (green), PVEL “*Phaseolus* Vellutina” (yellow), and PCANN “*Phaseolus* Cannellino” (red) separated on the right part of the plot, with the PON “*Phaseolus* Occhio Nero” variety (blue) on the left side of the plot (PC1 = 41.0% and PC2 = 32%), meaning that the last mentioned variety is very different in its metabolite composition from the other varieties.

Indeed, PON was qualitatively different in missing GABA and Pipecolic Acid; moreover, differences among the varieties were quantitatively observed comparing the intensities of the signals of the metabolites. Statistical analysis was performed by calculating the *p*-value of integral data obtained from the NMRProcFlow v1.4 software to understand the significance of the metabolites: leucine, lactic acid, alanine, methionine, citric acid, choline, pipecolic acid, sucrose, and trigonelline were the most significant metabolites (*p*-value < 0.05).

A targeted analysis using the PLS-DA projection method (Figure S15) was performed to further investigate the metabolites that most differentiated these varieties (PC1 = 89% and PC2 = 6%). The same integral data obtained using the NMRProcFlow software [76] (*vide supra*) for the *p*-value calculation of selected metabolites were used for the construction of the new data set. The score scatter plot confirmed the separation obtained with PCA, with PCANN and PCON really close between them. From the loading plot, the plot in which the variables are displayed, it was possible to observe that stachyose, choline, sucrose, methionine, pipecolic acid, and citric acid result as the metabolites responsible for the classification of the samples (Figure S16). In the VIP plot in component 1 (Figure S17a), sucrose, choline, pipecolic acid, methionine, and citric acid represent the metabolites that contribute to the discrimination of the samples in component 1 (VIP > 1), while in VIP plot component 2 (Figure S17b), stachyose, citric acid, sucrose, choline, pipecolic acid, and methionine represent the metabolites in the discrimination of the sample in component 2.

### 2.5. Phenolic Components and Antioxidant Activity

It is known that the colour of the seed coat of dried beans is determined by the presence and amount of flavonol glycosides, anthocyanins, and condensed tannins (proanthocyanidins) [11]. They constitute the polyphenolic fraction and are mainly responsible for the antioxidant activity of extracts.

Condensed tannins (CTs) (also referred to as proanthocyanidins) are flavonoid units linked by carbon–carbon bonds that are not susceptible to cleavage by hydrolysis. These compounds give the intense pink, red, purple, or blue colours of many flowers, fruits, and leaves and account for the astringent tastes of many fruits and wines. Tannins are naturally occurring, water-soluble, and are able to form complexes with proteins and polysaccharides [77]. CTs have been reported to exert multiple biological effects as well as exhibit anti-inflammatory, antioxidant, anticancer, and antibacterial activities [78]. It has been suggested that the activities of CTs not only depend on their antioxidant properties, as CTs are effective chelators of metal ions [79]. 

The Total Polyphenols Content (TPC), Total Flavonoids Content (TFC), and Condensed Tannins Content (CTs) were investigated to obtain a quantitative overview of the analytes in the batch samples.

The total polyphenols content (TPC) was determined by Folin-Ciocalteu assay [80]. Significant differences (*p* < 0.05) in TPC were found among the tested bean coats and the results are summarised in Table 2. The TPC content in four coats ranged from 1.35 to 0.47 mg GAE/g in the dry weight of whole beans. The overall highest TPC was found in Vellutina beans (PVEL) followed by Occhio Nero beans (PON, 1.11 mg GAE/g), both with a reddish toned coat. The kidney beans appeared as white and exhibited lower TPC, a trend in accordance with previous studies [81].

Flavonoids are the main phenolic compounds found in legumes [33]. The flavonoid concentration of the accessions ranged from 7.58 to 2.65 QE/g dw (Table 2). Coloured beans contain the highest concentration of flavonoids, as in *Phaseolus* Vellutina (PVEL, 7.58 mg QUE/g) and Occhio Nero (PON, 6.28 mg QUE/g), in accordance with their TPC content. A similar trend can be observed in the evaluation of condensed tannins (CTs) [82]. The highest concentration was observed in red beans PVEL (2.12 mg CAE/g dw) and PON (1.22 mg CAE/g dw), followed by the pale white sample PCON (0.21 mg CAE/g dw) and PCANN (0.08 mg CAE/g dw).

### 2.6. In Vitro Antioxidant Activity

Several studies have suggested a correlation between the content of phenolic compounds and the antioxidant properties of plant extracts. The antioxidant and antiradical properties of polyphenols result from the elimination of ROS through direct reactions, scavenging, or the reduction of free radicals to compounds with much lower reactivity [83]. Generally, the antioxidant potential follows the differences in the content of total phenols. The in vitro antioxidant activity of polar extracts was assessed using various in vitro assays, including the determination of DPPH scavenging activity, ABTS scavenging assay, and ferric-reducing antioxidant power (FRAP).

The potential antioxidant capacity of four samples was measured using 1,1-diphenyl2-picrylhydrazyl (DPPH) [80], ABTS (2,2′-azino-bis(3-ethylbenzothiazoline-6-sulfonic acid) [84], and ferric-reducing antioxidant power (FRAP) [85], model systems.

As shown in Table 3, the DPPH free radical scavenging activity (DPPH assay) of (PVEL) Vellutina and (PON) Occhio Nero flour extracts were higher with IC_50_ 1.43 mg/mL and IC_50_ 2.04 mg/mL, respectively, than (PCON) Controne (IC_50_ 8.49 mg/mL) and (PCANN) Cannellino (IC_50_ 9.70 mg/mL) extracts. A lower IC_50_ value indicates a higher antioxidant activity. The same trend was observed for the antioxidant capacity shown by brown seed coats (PVEL and PON) in the ABTS assay that was higher (4.55–4.05 μmol TE/mL) compared to non-dark seed coats PCON and PCANN (1.42 and 1.44 μmol TE/mL, respectively).

The ferric-reducing antioxidant power (FRAP) values for seed coat varieties ranged from 15.18 (PVEL) to 12.86 (PON) μmol TE/mL in whole red kidney beans; 4.27 (PCON) to 3.78 (PCANN) μmol TE/mL in the white varieties. The close relationship between TPC and antioxidant assay has been documented in several studies [43,45]. Pigmented beans showed higher antioxidant activity in all assays than pale white beans.

### 2.7. Correlation between Phenolic Compounds and the Antioxidant Activity of the Extract of Phaseolus Seeds

Pearson’s correlation coefficient (R) was used to describe the correlation between the total concentration of phenols (TPC), flavonoids (TFC), and condensed tannins content (CTs); antioxidant activity using different methods (DPPH, ABTS, and FRAP) is presented in Table 4.

A strong positive correlation was found between TPC and TFC (R = 1.00), indicating with high probability that the TPC is mainly responsible for the antioxidant activity in the samples. The most important positive correlations between TPC, TFC CTs with ABTS (R = 0.993, 0.994, 0.957, respectively), and FRAP assay (R = 0.985, 0.985, 0.963, respectively) indicate that the antioxidant capacity of extracts is due to the contribution of TPC, TFC, and CTs that are the dominant antioxidant in extracts. We also noticed a negative link between all phenolic components (TPC, TFC, and CTs) and DPPH assay with R = −0.979, −0.979, and −0.942, respectively. The ABTS method has a significant positive correlation with the FRAP method (R = 0.988), while a negative correlation was evidenced between the DPPH method and both ABTS (R = −0.991) and FRAP (R = −0.997).

The results obtained indicate that the phenolic-rich fractions of polar extracts have high antioxidant potential and can be used in the development of health-benefitting functional foods through daily inclusion in the human diet.

### 2.8. Antifulgal Activity

The antifungal activity of the four aqueous extracts against the growth of *S. rolfsii* sclerotia was carried out in vitro. The hydroalcoholic extracts of the four commercial varieties of *Phaseolus* under investigation, i.e., Cannellino (PCANN), Controne (PCON), Vellutina (PVEL), and Occhio Nero (PON), were used at different concentrations, ranging from 0 to 37.5 mg, as described in Material and Methods (vide infra). The results showed no antifungal activity for all beans at concentrations ranging from 0 to 8.75 mg of extracts, while, for three of them, even at 37.5 mg of hydroalcoholic extract, they grew at the same rate as the negative control. Only the PON (Occhio Nero) extract showed antifungal activity, inhibiting halo growth by 75% at 37.5 mg. The positive control, with Thiram in the well, showed no fungal growth.

In recent literature, it has been reported that increasing the concentration of organic acids (citric, lactic, malic acid) leads to a visible inhibition of mycelial growth [86]. Notably, malic acid has also demonstrated antimicrobial activity, causing significant damage in the cell cytoplasm [87].

The result exclusively observed in the PON sample could be related to the higher concentration of malic acid detected in the seeds compared to the other varieties. However, it cannot be excluded that, in such a complex system of metabolites, synergistic effects with other minor components may occur.

## 3. Materials and Methods

### 3.1. General Information

For the chemical fingerprinting analysis, we used NMR-based metabolomics. NMR is an effective and non-destructive analytical tool by which to identify the structure of molecules and is usefully applied in the determination of food quality, geographical origin, ecological studies, or plant specimen classification [88,89,90]. Untargeted proton nuclear magnetic resonance (^1^H NMR) methodologies were applied to the ground beans to obtain flour [91]. Because of the richness of information, which often results in high spectral complexity, it requires multivariate data analysis to provide qualitative and quantitative metabolite information to compare or monitor the level of metabolite composition usually found in complex mixtures. Chemometrics, as mathematical tools, are widely used for the discrimination, authentication, and quality assessment of food, herbal medicine, and crops.

Applying Principal Component Analysis (PCA) to NMR data allowed us to highlight and summarise the similarities and differences between commercial bean seeds. Many local genotypes are still cultivated in marginal areas of Italy, and in this context, the consumption of local ecotypes may afford a higher market value. In Italy, such products are labelled by geographic provenance (IGP) indication.

**Figure 8:**
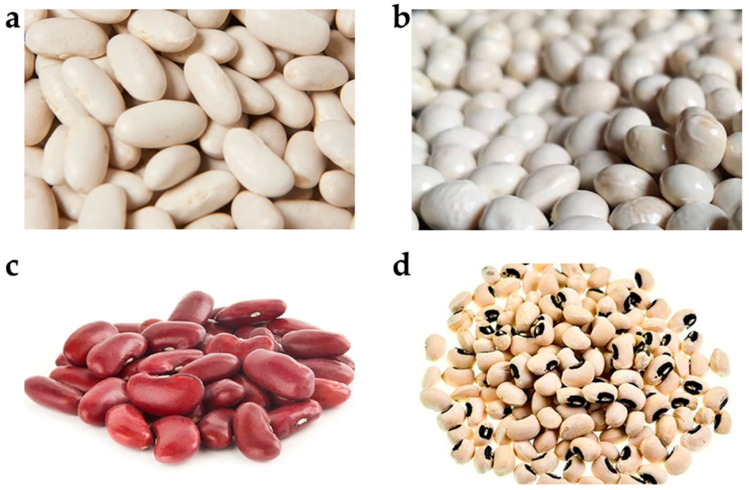
Commercial beans selected for the analysis: (**a**) *Phaseolus* Cannellino (PCANN), (**b**) *Phaseolus* Controne (PCON); (**c**) *Phaseolus* Vellutina (PVEL), and (**d**) *Phaseolus* Occhio Nero (PON).

The bean seeds sampled in this study were purchased in the specialised market in Naples (Italy). All samples were Italian commercial varieties from the Umbria and Campania regions. The seeds had different colours (Figure 8), from pale white (*Phaseolus* Cannellino, PCANN, and *Phaseolus* Controne, PCON) to red (*Phaseolus* Vellutina, PVEL) and to a reddish tone with dark spots (*Phaseolus* Occhio Nero, PON) and different shapes (Figure 8).

### 3.2. Standards and Reagents

Dichloromethane (Sigma-Aldrich, Milan, Italy), Methanol (Sigma-Aldrich, Milan, Italy), and Water milliQ were used for the extraction for NMR analysis. Deuterium oxide (99.95% D, Sigma-Aldrich, Milan, Italy) and TMSP as a reference in D_2_O (0.75 wt. % sodium salt, 99.9% D, Sigma-Aldrich, Milan, Italy) were used for NMR analysis of the polar extract; the non-polar extract was prepared in Chloroform-d (99.8%, Sigma-Aldrich, Milan Italy) using TMS as a reference in CDCl_3_ (0.03% *v*/*v*, 99,8% D, Sigma-Aldrich, Milan, Italy).

DPPH radical, ascorbic acid, (+)-Catechin, Folin-Ciocalteu reagent, gallic acid, sodium carbonate, 2,4,6-tri(2-pyridyl)-s-triazine (TPTZ), 6-hydroxy-2,5,7,8-tetramethlchroman-2-carboxylic acid (Trolox), and 3,3′,4′,5,7-Pentahydroxyflavone dehydrate (Quercetin) were obtained from Sigma Chemical Co. (St. Louis, MO, USA). All solvents used for extraction were purchased from VWR Intl. (West Chester, PA, USA). All other chemicals were of analytical grade.

### 3.3. Plant Material and Extraction Procedure

Plant Material. The bean seeds sampled in this study were purchased in the specialised market in Naples (Italy). All samples were Italian commercial varieties: Cannellino bean (Umbria Region), Controne bean (Campania Region), Vellutina bean (Umbria Region), and Occhio Nero bean (Umbria Region).

Seeds of commercial *Phaseolus* were triturated in mortar, obtaining a fine powder. Five aliquots (1.5 g each) were extracted with CH_2_Cl_2_/H_2_O/MeOH (2:1:1) according to the procedure reported by de Falco et al. [92] for 1 h at room temperature on a magnetic plate. The polar phase (at the top) and non-polar phase (at the bottom) were accurately separated, with particular attention to discard the interphase, which was extracted a second time for 30 min. Both polar and non-polar extracts were centrifuged (3000 rpm, 30 min, room temperature) and dried with the Rotavapor at 30 °C. Extracts were then transferred into Eppendorf tubes, dried using a Speed-Vac, and lyophilised for 2 days until NMR analysis.

### 3.4. Nuclear Magnetic Resonance (NMR) Experiments

NMR experiments were acquired on a Bruker DRX-600 spectrometer (Bruker BioSpinGmBH, Rheinstetten, Germany) at 600 MHz. The acquisition parameters [61] were: FID size = 64 K, spectral width = 15.00 ppm, receiver gain = 90.5, scans = 512, and number of dummy scans = 4. Data acquisition for the polar extract was also achieved by using a Carr-Purcell-Meiboom-Gill (CPMG) pulse sequence (Bruker 1D cpmgpr1d). This strategy was applied to separate the signals of low-weight metabolites from macromolecules [93]. The analyses were based on 1D ^1^H-NMR and bidimensional experiments: homonuclear correlation spectroscopy (2D ^1^H–^1^H COSY), heteronuclear single quantum correlations (2D ^1^H–^13^C HSQC), and heteronuclear multiple bond correlation (2D ^1^H–^13^C HMBC). COSY measurements were made with 4 k × 256, a spectral width of 12 in either dimension, 24 scans, and a 2 s relaxation delay. For HSQC, 4 k × 256 were acquired with 32 scans, a spectral width of 11, and 150 for the ^1^H and ^13^C dimensions, respectively, and a 1 s relaxation delay. The parameters for HMBC were 4 k × 256, 48 scans, a spectral width of 11 and 230 for the ^1^H and ^13^C dimensions, respectively, and a 1 s relaxation delay.

The 1D and 2D NMR analyses were carried out using a Bruker 600 MHz spectrometer operating at 298 K.

### 3.5. Sample Preparation and NMR Analyses

An amount of 7mg of dried and lyophilised polar extract was dissolved in 450 µL of D_2_O (99.95%) containing phosphate buffer saline (PBS, pH 7.4), and 50 µL of TMSP 1 mM (0.1 mM final concentration) was added as a reference [94]. An amount of 7mg of non-polar extract was diluted in 450 µL of CDCl_3_ (99.8%), and 50 µL of TMS 0.03% *v*/*v* as a reference was added. The samples (500 µL each) were transferred into 5-mm NMR tubes for analysis.

The processing of NMR spectral data was performed on TOPSPIN 3.5 software with manual phase correction, baseline correction, and calibration, adjusting the TMSP shift signal to 0.00 ppm.

### 3.6. Metabolite Identification

Metabolite profiling was assigned by literature data, with standard samples available in the laboratory and by comparison with the Human Metabolome Data Base (HMDB; http://hmdb.ca/, accessed on 31 October 2023) and Chenomx software version 9.0. Signal assignments were confirmed by recording and analysing 1D and 2D NMR experiments. These were ^13^C spectroscopy (1D), Homonuclear Correlation Spectroscopy (2D, ^1^H–^1^H COSY), Heteronuclear Single Quantum Correlations (2D, ^1^H–^13^C HSQC), and Heteronuclear Multiple Bond Correlation (2D, ^1^H–^13^C HMBC).

### 3.7. Quantification of Metabolites (qNMR)

The spectra of all polar extracts were imported to NMRProcFlow software [76] (https://nmrprocflow.org/, accessed on 15 November 2023), and the most resolved and non-overlapped signals of metabolites were quantified using the following processing: calibration on TMSP signal from 0.1 to −0.1 ppm, soft global baseline correction, alignment of signals, qNMR (quantitative NMR) baseline correction of selected signals, and the bucketing “Variable Size Buckets” was applied to the selected signals. A range of ppm from 0.1 to −0.1 was indicated as a reference, and the Microsoft Excel workbook was generated and completed with the right parameters for quantification: the amount of extract, the volume of the sample, the molecular weight of the metabolites, and the number of protons related to the selected signals. Integral data that the software calculates and uses for quantification were generated in the same Excel workbook and were collected for targeted multivariate and statistical analyses.

### 3.8. Chemometric Analysis

The NMR data of polar extracts were analysed using untargeted multivariate analysis through Principal Component Analysis (PCA) to assess variations in the samples’ chemical profile [95]. Prior to the PCA, spectra were processed for the multivariate analysis in a way similar to [96]: the baseline of the spectra was corrected, and the TMSP shift signal was adjusted to 0.00 ppm for calibration and alignment of the signals. Then, spectra were stacked, each NMR spectrum was reduced to integrated buckets (binning) of an equal width of 0.01 ppm in the range of *δ* 10.0–0.5, and the spectra were normalised to the total area. Spectral region 4.67–4.83 ppm was excluded from the analyses to eliminate the effects of water. In this way, a data set with N observation (20 analysed samples, 4 varieties extracted 5 times) and X variables (X H^1^ NMR signals) was imported to the SIMCA-P software, version 17 (Umetrics, Umea, Sweden). The quality of the model was determined by R2 and Q2 values (R2X[1]: 0.41, R2X[2]: 0.32).

Integral data obtained from NMRProcFlow software were used for the *p*-value calculation of selected metabolites in order to determine their significant difference levels (*p*-value < 0.05) and for a targeted multivariate analysis in order to understand the metabolites that most contribute to the differentiation of the bean varieties. In this case, Partial-Least-Squares Discriminant-Analysis (PLS-DA) and Variable Importance in Projection (VIP) analysis were performed with SIMCA software with the goal of investigating and disclosing the most important metabolites that contribute to the differentiation of the varieties. The validation of the model was conducted using permutation tests. Also, in this case, the quality of the model was determined by R2 and Q2 values (R2X[1]: 0.89, R2X[2]: 0.06). For the multivariate analysis, both PCA and PLS-DA Pareto-scale were used: this is recommended in NMR-based metabolomics due to the noise and data reliability [97].

### 3.9. Extraction of Bean Samples

A sample of 0.45 g of powder was extracted in a capped centrifuge tube with 9 mL of methanol (80% *v*/*v*). Hence, samples were sonicated using an ultrasonic bath (Sonica, Ultrasonic cleaner, Milan, Italy) for 45 min, incubated at room temperature (25 °C) in the dark for 15 min and subsequently centrifuged for 15 min at 5000 rpm. The supernatant was removed into new tubes, and extracts were stored at 4 °C in the dark until further analysis.

#### 3.9.1. Folin-Ciocalteu Assay: Determination of Total Phenolic, Flavonoid Content and Condensed Tannins Content

The total phenolic content was determined using the Folin–Ciocalteu method, described by Heimler et al. [80]. To 1 mL of the suitably diluted sample extract, 0.5 mL of the Folin-Ciocalteu (previously diluted with water 1:10 *v*/*v*) reagent was added. The mixture was kept for 5 min, and then 3 mL of Na_2_CO_3_ (7%; *w*/*v*) was added. The final volume was adjusted to 5 mL with water. A blue coloration developed, and after 1 h, the absorbance was read at 765 nm (Shimadzu UV-1601 spectrophotometer, Shimadzu, Kyoto, Japan) against the blank consisting of all reagents and solvents without test compounds. The total phenolic content was expressed as gallic acid equivalents (mg GAE/g sample) through the calibration curve (1–10 μg/mL) of gallic acid.

#### 3.9.2. Total Flavonoid Content (TFC)

The total flavonoid content in the extracts was evaluated according to the method [80].

Briefly, an aliquot (1 mL) of the bean extract was mixed with distilled water (0.270 mL), followed by adding NaNO_2_ solution (5%, 80 μL). After 6 min, an AlCl_3_ × 6 H_2_O solution (10%, 150 μL) was added and allowed to stand for another 5 min, in the dark, before adding NaOH solution (1.0 M, 500 μL). The mixture was brought to 2.5 mL with distilled water and mixed well. The absorbance was immediately measured against the blank (the same mixture without the sample) at 510 nm (Shimadzu UV-1601 spectrophotometer). The results were calculated and expressed as mg of (+)-catechin equivalents (mg CAE/g DM sample) using the calibration curve of (+)-catechin. The linearity range of the calibration curve was 2 to 50 μg/mL for (+)-catechin and 2 to 100 μg/mL for quercetin.

#### 3.9.3. Condensed Tannins (TCT)

The analysis of total condensed tannins was determined following the procedure reported by Heil et al. [82].

Briefly, 100 μL of methanol extract was thoroughly mixed with 1 mL of 4-(dimethylamino)cinnamaldehyde (DMCA) (0.1% of DMCA in methanol-HCl (9:1 *v*/*v*)) within a cuvette. After 5 min of incubation at room temperature, the absorbance of each sample was measured at 640 nm (Shimadzu UV-1601 spectrophotometer). The amount of condensed tannin was calculated and expressed as mg (+)-catechin equivalents (mg CAE/g sample) through a calibration curve of (+)-catechin. The linearity range of the calibration curve was 1 to 15 μg/mL.

### 3.10. Antioxidant Capacity

The antioxidant capacity was determined with three different methods: DPPH• (1,1-Diphenyl-2-picrylhydrazyl) radical scavenging activity, ABTS (2,2′-Azino-bis(3-ethylbenzthiazoline-6-sulfonic acid), and FRAP (Ferric-Reducing Antioxidant Power) procedures.

#### 3.10.1. DPPH Radical Scavenging Activity

The free radical scavenging capacity of beans extract was evaluated by DPPH assay according to the procedure reported by Heimler et al. [80] and slightly modified.

An amount of 1 mL of the sample solution, suitably diluted with methanol (80% *v*/*v*), was added to 1 mL of a freshly prepared DPPH• methanolic solution (27 μg/mL) and 3 mL of methanol (80% *v*/*v*). The reaction mixture was kept in the dark for 30 min; when the DPPH radical reacts with an antioxidant compound, it is reduced and changes colour. The colour changes were read as absorbance at 517 nm (Shimadzu UV-1601 spectrophotometer) using methanol (80% *v*/*v*) as a blank. Ascorbic acid was used as a positive control. The antioxidant activity is expressed in terms of IC_50_, the value denotes the extract concentration (mg/mL), which is required to scavenge 50% of DPPH free radicals. This value was calculated by linear regression analysis of the dose–response curve (range 0.25–2.5 mg/mL), which was obtained by plotting the radical scavenging activity against extract concentration.

To calculate the IC_50_, different percentages of inhibition were determined as follows:

% Radical Scavenging Activity = [1 − (A_sample_/A_control_)] ∗ 100 (1)

where A_control_ is the absorption of the 1 mL of DPPH• and 4 mL of methanol (80% *v*/*v*) and A_sample_ is the absorption of the sample.

#### 3.10.2. ABTS Radical Scavenging Activity

The antioxidant capacity of bean methanolic extracts was determined according to [84] with some modifications. The ABTS radical cation (ABTS•+) was produced by reacting an ABTS solution (7 mM) with potassium persulfate (2.45 mM) for 16 h, in the dark, at room temperature (1:1 *v*/*v*). The solution with the radical was diluted in methanol (80% *v*/*v*) to an absorbance of 0.70 ± 0.05 to 734 nm. For the test, 150 μL of the extract was mixed with 1350 μL of the reaction solution, placed in incubation in darkness for 30 min, and the absorbance was read at 734 nm (Shimadzu UV-1601 spectrophotometer). The ability of the bean sample to scavenge ABTS radicals was calculated as % inhibition by the following equation:

% ABTS Inhibition = [1 − (A_sample_/A_control_)] ∗ 100 (2)

where Abs_control_ is the absorbance of the 150 μL of methanol with 1350 μL of ABTS.

The absorbance of the reaction samples was compared to that of the Trolox standard, and the results were expressed as μmol Trolox equivalents per g dry weight of sample (μmol TE/g DW).

#### 3.10.3. FRAP Assay

The FRAP assay was performed as previously described by Benzie et al. [85], with modifications. The working FRAP reagent was prepared by mixing acetate buffer (300 mM, pH 3.6) with TPTZ (2,4,6-tripyridyl-s triazine) solution (10 mM in 40 mM HCl) and with FeCl_3_ × 6 H_2_O (20 mM) in a ratio 10:1:1. A total of 200 μL of the extract was reacted with 1.8 mL of FRAP and incubated at 37 °C during 30 min. The absorbance was measured at 593 nm (Shimadzu UV-1601 spectrophotometer). The in vitro antioxidant activity quantification was performed using a standard Trolox curve in a concentration range between 5 to 40 mM. The results were expressed in μmol of Trolox equivalents per gram of dry weight (μmol TE/g 1 DM).

#### 3.10.4. Statistical Analysis

Assays were performed in triplicate, and results are shown as mean ± standard deviation (SD). Correlations between TPC, TFC, and TCT, and the antioxidant capacity by different methods (DPPH, ABTS, and FRAP) were determined using Pearson’s correlation coefficient (R). A statistical significance of *p* < 0.05 was considered to be significant.

Correlation analyses (Table 4) between phenolic content and antioxidant activities among all bean samples were performed and exhibited significant (*p* ** < 0.01 and *p* * < 0.05) linear correlations. Pearson’s correlation coefficient (R) and linear regression, assessment of repeatability of average and relative standard deviation was performed using Microsoft Excel 2016 software (Microsoft, Washington, DC, USA).

### 3.11. Determination of Antifungal Activity

Dehydrated aqueous fractions of commercial varieties, i.e., Cannellino (PCANN), Controne (PCON), Vellutina (PVEL), and Occhio Nero (PON), were resuspended in 200 µL of distillated water obtaining a solution with a concentration of 0.25, 0.25, 0.26, and 0.36 mg/µL, respectively. Subsequently, 3.75, 8.75, and 37.5 mg of beans’ aqueous solutions were added into 3 mL of PDA medium (Oxoid™, Basingstoke, UK), and autoclaved. Sterilised medium containing the beans’ aqueous extracts was poured off into 12-well multi dishes (Corning™, Somerville, MA, USA). The sclerotia of *S. rolfsii*, identified as described by Falasca et al. [98], were used as inoculum. The growth of mycelia was monitored for 72 h at 25 °C. The positive controls for the antifungal activity were carried out using PDA added with Thiram (Tetrasar 50, powder, Isagro Srl, Aprilia, Italy) al 80 µg/mL. The tests were conducted in triplicate.

## 4. Conclusions

The seeds of *Phaseolus* and other legumes are a source of phytochemicals that have received increased attention for their human health benefits. Several of these actions are mainly related to non-nutritional compounds such as polyphenols, protease inhibitors, carbohydrates, and saponins. The results presented show that each local variety has its own chemical profile responsible for the nutritional and sensory properties linked to its components, which could be used as ingredients for health products, functional foods, dietary supplements, and cosmetics. For instance, the brown *P.* Vellutina (PVEL) and *P.* Occhio Nero (PON) cultivars show the highest concentration of polyphenols with recognised antioxidant, anti-inflammatory, and antidiabetic properties. The minor polyphenol components still need to be investigated in depth as the sensitivity of NMR spectroscopy does not allow the lower concentrations to be determined. Therefore, further in-depth studies can be carried out using mass spectrometry. The results of commercial *Phaseolus* Italian varieties suggest that this experimental workflow could help trace and/or complete the fingerprint of other partially unexplored varieties and protect the peculiarities of regional foods for obtaining geographical provenance indications, promoting rural and agricultural activity, and enhancing biodiversity. The data reported could be useful for national and international information systems to enhance the agri-food sectors by providing producers and industries with accurate information on the characteristics of local products to be marketed.

## Figures and Tables

**Table 1:** ^1^H NMR chemical shifts, assignment, and multiplicity at 600 MHz in D_2_O of the metabolites identified in all analysed polar and lipophilic extracts of commercial beans.

Compound	Metabolite	Assignment	^1^H (ppm) Multiplicity [*J* (Hz)]	^13^C (ppm)	PON Occhio Nero
***Aminoacids***					
**1**	Alanine (Ala)	β-CH_3_	1.48 (d, 7) #	16.00	x
**2**	Arginine (Arg)	β,β′-CH_2_ γ-CH_2_ δ-CH_2_	1.91 m, 1.73 m 1.66 m # 3.23 (t, 7)		
**3**	Asparagine (Asn)	β,β′-CH_2_	2.95 * 2.86 (dd, 7.3, 16.8)	34.3	2.96 (dd, 4.3, 12.6) 2.87 (dd, 7.4, 16.8)
**4**	Aspartate (Asp)	β,β′-CH_2_	3.02 * 2.96 *	35.6	3.01 (dd, 4.8, 17.3) # 2.95 *
**5**	γ-aminobutyrate (GABA)	α-CH_2_ γ-CH_2_	2.30 (t,7.3) 2.99 (t,7.3)	34.4 35.9	NOT PRESENT
**6**	Glutamate (Glu)	β,β′-CH_2_ γ-CH_2_	2.07 *; 2.14 * 2.36 m		x
**7**	Isoleucine (Ile)	γ-CH_3_	1.01 (d, 7.3) #	15.2	x
**8**	Leucine (Leu)	δ-CH_3_ δ′-CH_3_	0.93 (d, 6.5) 0.90 (d, 6.5) #	22.4 20.6	
**9**	Methionine (Met)	α-CH β-CH_2_ γ-CH_2_ -S-CH_3_	2.52 * 2.38 * 2.17 * 2.14 s #	33.4 26.6 14.5	2.52 t
**10**	Phenylalanine (Phe)	2,6-CH 3,5-CH	7.32 (d, 7.0) 7.42 (m)	- -	x
**11**	Proline (Pro)	γ-CH_2_ β-CH_2_	2.00 m 2.08 m	- -	
**12**	Threonine (Thr)	γ-CH_3_ α-CH β-CH	1.33 (d, 6.0) # 3.51 * 4.23 *	19.5	x
**13**	Tyrosine (Tyr)	2,6-CH 3,5-CH	7.18 (d, 7.0) 6.89 (d, 7.0) #	- -	7.16 6.90
**14**	Tryptophan (Trp)	7-CH 4-CH 2-CH α-CH β-CH_2_	7.54 (d, 8.0) # 7.74 (d, 8.0) 7.32 * 4.38 * 3.29 *		x
**15**	Valine (Val)	γ-CH_3_ γ′-CH_3_	0.99 (d, 7) 1.05 (d, 7) #	16.5 17.8	x
***Carbohydrates***					
**16**	Sucrose	CH-1 (Glc) CH-2 (Glc) CH-3 (Glc) CH-4 (Glc) CH-3′ (Fru) CH-4′ (Fru) CH_2_-1′(Fru) CH-6′ (Fru)	5.42 (d, 4.0) 3.57 3.76 (t) 3.47 (t, 9.6) # 4.23 (d, 9) 4.05 (t) 3.68 3.78 *	92.7 69.0 76.3 74.0 61.6	x
**17**	Raffinose	CH-1 (Glc) CH-1 (Gal) CH-3 (Fru) CH-3 (Glc) CH-5 (Glc) CH-5 (Gal)	5.42 (d, 4.0) 5.00 (d, 4) 4.22 (d, 9) 3.77 (t) 4.04 * 4.01 *	92.7 98.2 74.1 72.6 73.8 70.7	x
**18**	Stachyose	CH-1 (Glc) CH-1 (Gal-T and Gal-I) CH-3 (Fru) CH_2_-1 (Fru)	5.44 (d, 4) # 5.00 m 4.21 (d, 9) 3.68	92.0 98.3 76.3 61.6	x
***Organic acids***					
**19**	Citric acid	α,γ-CH α’,γ′-CH	2.58 (d, 16.0) # 2.70 (d, 16.0)	44.5	x
**20**	Formic acid	-CH	8.44 s #	-	x
**21**	Lactic acid	-CH_3_	1.25 (d, 6.2) #	22.8	x
**22**	Malic acid	α-CH β-CH	4.30 (dd, *) # 2.35 (dd, *)	70.3 33.8	x
**23**	Pipecolic acid (Pip)	CH-3,4,5 CH-3′,4′,5′ CH-6 CH-6′ CH-2 N-H	2.22–1.67 m 1.73–1.87 m 3.00 (td, 12.5,3.2) 3.41 dd # 3.59 (dd, *) 2.17 m	43.5 59.0	NOT PRESENT
***Other components***					
**24**	Adenosine (Ade)	CH-1′ CH-2 CH-8	6.07 (d,5.5) 8.27 s 8.35 s #		x
**25**	Choline (Cho)	-N(CH_3_)_3_^+^	3.21 s #	53.6	x
**26**	Guanosine (Gua)	CH-8	5.92		x
**27**	Uridine (Uri)	CH-6	5.91 d 7.87 d #		x
**28**	Trigonelline (Tri)	H-2 H-4,6 H-5 -N-CH_3_	9.13 s # 8.83 m 8.09 m 4.44 s	145.7 145.5 127.7 48.2	x
**29**	p-coumaric acid derivative	2,6-CH 3,5-CH CH=CH	7.62 (d, 8.8) 6.97 (d,8.8) 7.79–6.51 (d, 16.1)		x
***Lipophilic extracts***					
	Fatty acids				
		ω_1_-CH_3_	0.88 (t,7.5)	13.5	x
**30**	α-linolenic acid	ω_3_-CH_3_	0.97 (t, 7.5)	14.2	x
		-(CH_2_)_n_-	1.25, 1.30	29.0	x
		-CH_2_-CH_2_COO^−^	1.61	24.4	x
		allylic-CH_2_	2.05 m	27.1	x
		-CH_2_COO^−^	2.31	34–35	x
**31**	α-linolenic acid	bis-allylic -CH_2_	2.81 m	24.9	x
**32**	α-linoleic acid	bis-allylic -CH_2_	2.77 m	25.1	x
***Glicerol***					
**TAG** (glycerol backbone)	*sn*-1 and *sn*-3	-CH_2_-O-CO-	4.29 (dd, 11.9, 4.4) 4.15 (dd, 11.9, 5.7)	61.8	x
	*sn*-2	2′-CH-O-CO-	5.27 m	68.1	x
	*sn*-1,2/2,3 DAG	OH-CH_2_-CH-	3.74 m	62.2	x
	*sn*-1,2 DAG	1′ *b*-CH_2_-O-CO-	4.36 dd		
		-CH=CH-	5.37 m	127.6 129.6	x

* Signal overlapped; # Signals used for quantification; TAG: Triacylglycerides; DAG: Diacylglyceride.

**Table 2:** Total Phenolic, Flavonoid contents and Condensed Tannins of methanolic extracts in different varieties of beans.

SAMPLE	Total Phenolic	Flavonoids	Condensed Tannins
	(mg GAE/g d.w.)	(mg QUE/g d.w.)	(mg CAE/g d.w.)
**PVEL**	1.35 ± 0.05	7.58 ± 0.13	2.12 ± 0.02
**PON**	1.11 ± 0.02	6.28 ± 0.40	1.22 ± 0.01
**PCON**	0.47 ± 0.01	2.65 ± 0.05	0.21 ± 0.01
**PCANN**	0.48 ± 0.02	2.72 ± 0.10	0.08 ± 0.01

GAE: Gallic acid equivalents; QUE: Quercetin equivalents; CAE: Catechin equivalents. Each value is a mean ± SD of triplicate analysis. *Phaseolus* Cannellino (PCANN), *Phaseolus* Controne (PCON), *Phaseolus* Vellutina (PVEL), and *Phaseolus* Occhio Nero (PON).

**Table 3:** Total antioxidant capacity using DPPH, ABTS, and FRAP methods.

SAMPLE	DPPH	ABTS	FRAP
	IC_50_ (mg/mL)	(μmol TE/mL)	(μmol TE/mL)
**PVEL**	1.43 ± 0.06	0.56 ± 0.06	15.18 ± 0.53
**PON**	2.04 ± 0.09	0.50 ± 0.04	12.86 ± 0.53
**PCON**	8.49 ± 0.23	0.17 ± 0.02	4.27 ± 0.23
**PCANN**	9.70 ± 0.37	0.18 ± 0.01	3.78 ± 0.15

IC_50_: concentration of the extract that inhibits 50% of the radical activity. TE: Trolox equivalents. Each value is a mean ± SD of triplicate analysis. *Phaseolus* Cannellino (PCANN), *Phaseolus* Controne (PCON), *Phaseolus* Vellutina (PVEL), and *Phaseolus* Occhio Nero (PON).

**Table 4:** Pearson’s correlation coefficients (R) between antioxidant capacity and metabolite groups.

Correlation Coefficients (R)	Total Phenolic	Flavonoids	Condensed Tannin	DPPH	ABTS	FRAP
Total Phenolic	1					
Flavonoids	1.000 **	1				
Condensed Tannin	0.983 *	0.982 *	1			
DPPH	−0.979 *	−0.979 *	−0.942 *	1		
ABTS	0.993 **	0.994 **	0.957 *	−0.991 **	1	
FRAP	0.985 *	0.985 *	0.963 *	−0.997 **	0.988 *	1

** Correlation is significant at the 0.01 level. * Correlation is significant at the 0.05 level. *Phaseolus* Cannellino (PCANN), *Phaseolus* Controne (PCON), *Phaseolus* Vellutina (PVEL), and *Phaseolus* Occhio Nero (PON).

## Data Availability

Data are contained within the article and Supplementary Materials.

## Supplementary Materials

The following supporting information can be downloaded at: https://www.mdpi.com/article/10.3390/plants13020227/s1, Figure S1. ^1^H-NMR spectrum in D_2_O of polar extract of *Phaseolus* Cannellino (PCANN) at 600 MHz; Figure S2. ^1^H-NMR spectrum in D_2_O of polar extract of *Phaseolus* Controne (PCON) at 600 MHz; Figure S3. ^1^H-NMR spectrum in D_2_O of polar extract of *Phaseolus* Occhio Nero (PON) at 600 MHz; Figure S4. ^1^H-^1^H-COSY NMR spectrum in D_2_O of polar extract of *Phaseolus* Vellutina (PVEL) at 600 MHz; Figure S5. HSQC spectrum in D_2_O of polar extract of *Phaseolus* Vellutina (PVEL) at 600 MHz; Figure S6. HMBC spectrum in D_2_O of polar extract of *Phaseolus* Vellutina (PVEL) at 600 MHz; Figure S7. Chemical structure of selected metabolites of both polar and apolar extract of *Phaseolus* seeds, namely Tyrosine (**13**, Tyr), Tryptophan (**14**, Trp), Pipecolic acid (**23**, Pip) and Trigonelline (**28**, Tri); Figure S8. ^1^H-NMR spectrum in CDCl_3_ of apolar extract of *Phaseolus* Cannellino (PCANN) at 600 MHz; Figure S9. ^1^H-NMR spectrum in CDCl_3_ of apolar extract of *Phaseolus* Controne (PCON) at 600 MHz; Figure S10. ^1^H-NMR spectrum in CDCl_3_ of apolar extract of *Phaseolus* Occhio Nero (PON) at 600 MHz; Figure S11. ^1^H-^1^H-COSY NMR spectrum spectrum in CDCl_3_ of apolar extract of *Phaseolus* Vellutina (PVEL) at 600 MHz; Figure S12. HSQC spectrum in CDCl_3_ of apolar extract of *Phaseolus* Vellutina (PVEL) at 600 MHz; Figure S13. HMBC spectrum in CDCl_3_ of apolar extract of *Phaseolus* Vellutina (PVEL) at 600 MHz; Figure S14. 2D representation of Principal Component Analysis (PCA) of polar extracts of bean varieties; Figure S15. Partial Least Square—Discriminant Analysis (PLS-DA) of the selected metabolites of polar extracts; Figure S16. Loading Plot of PLS-DA; Figure S17. (a) Variable Importance in Projection on component 1. (b) Variable Importance in Projection on component 2.
