# Supplementary material for: NMR Metabolomics and Chemometrics of Commercial Varieties of Phaseolus vulgaris L. Seeds from Italy and In Vitro Antioxidant and Antifungal Activity
Source: Plants (Basel). 2024 Jan 13;13(2):227. doi: 10.3390/plants13020227 (PMC10820859; doi:10.3390/plants13020227)
Supplement: Supplementary file 1 [file plants-13-00227-s001.zip › plants-2786544-supplementary.pdf]

SUPPORTING INFORMATION

NMR Metabolomics and Chemometrics of Commercial Varieties of *Phaseolus vulgaris* L. Seeds from Italy and In Vitro Antioxidant and Antifungal Activity

Vadym Samukha [1,†], Francesca Fantasma [1,†], Gilda D'Urso [2], Claudio Caprari [1], Vincenzo De Felice [1], Gabriella Saviano [1], Gianluigi Lauro [2], Agostino Casapullo [2], Maria Giovanna Chini [1,*], Giuseppe Bifulco [2,*] and Maria Iorizzi [1]

[1] Department of Biosciences and Territory, University of Molise, Contrada Fonte Lappone, 86090 Isernia, Italy; v.samukha@studenti.unimol.it (V.S.); fantasma@unimol.it (F.F.); claudio.caprari@unimol.it (C.C.); defelice@unimol.it (V.D.F.); saviano@unimol.it (G.S.); iorizzi@unimol.it (M.I.)
[2] Department of Pharmacy, University of Salerno, Via Giovanni Paolo II 132, 84084 Salerno, Italy; gidurso@unisa.it (G.D.); glauro@unisa.it (G.L.); casapullo@unisa.it (A.C.)
* Correspondence: mariagiovanna.chini@unimol.it (M.G.C.); bifulco@unisa.it (G.B.); Tel.: +39-0874404132 (M.G.C.); +39-089969741 (G.B.)
† These authors contributed equally to this work.

Table of contents

Figure S1. ^{1}H-NMR spectrum in D_2O of polar extract of *Phaseolus* Cannellino (PCANN) at 600 MHz. 3

Figure S2. ^{1}H-NMR spectrum in D_2O of polar extract of *Phaseolus* Controne (PCON) at 600 MHz. .. 3

Figure S3. ^{1}H-NMR spectrum in D_2O of polar extract of *Phaseolus* Occhio Nero (PON) at 600 MHz. 4

Figure S4. ^{1}H-^{1}H-COSY NMR spectrum in D_2O of polar extract of *Phaseolus* Vellutina (PVEL) at 600 MHz. 4

Figure S5. HSQC spectrum in D_2O of polar extract of *Phaseolus* Vellutina (PVEL) at 600 MHz. 5

Figure S6. HMBC spectrum in D_2O of polar extract of *Phaseolus* Vellutina (PVEL) at 600 MHz. 5

Figure S7. Chemical structure of selected metabolites of both polar and apolar extract of *Phaseolus* seeds, namely Tyrosine (13, Tyr), Tryptophan (14, Trp), Pipecolic acid (23, Pip) and Trigonelline (28, Tri). 6

Figure S8. ^{1}H-NMR spectrum in $CDCl_3$ of apolar extract of *Phaseolus* Cannellino (PCANN) at 600 MHz. 6

Figure S9. ^{1}H-NMR spectrum in $CDCl_3$ of apolar extract of *Phaseolus* Controne (PCON) at 600 MHz. 7

Figure S10. ^{1}H-NMR spectrum in $CDCl_3$ of apolar extract of *Phaseolus* Occhio Nero (PON) at 600 MHz. 7

Figure S11. ^{1}H-^{1}H-COSY NMR spectrum in $CDCl_3$ of apolar extract of *Phaseolus* Vellutina (PVEL) at 600 MHz. 8

Figure S12. HSQC spectrum in $CDCl_3$ of apolar extract of *Phaseolus* Vellutina (PVEL) at 600 MHz . 8

Figure S13. HMBC spectrum in $CDCl_3$ of apolar extract of *Phaseolus* Vellutina (PVEL) at 600 MHz. 9

Figure S14. 2D representation of Principal Component Analysis (PCA) of polar extracts of bean varieties. PCANN (red), PCON (green), PVEL (yellow) and PON (blue) are separated in the score plot. 9

Figure S15. Partial Least Square – Discriminant Analysis (PLS-DA) of the selected metabolites of polar extracts. 10

Figure S16. Loading Plot of PLS-DA. 10

Figure S17. a) Variable Importance in Projection on component 1. b) Variable Importance in Projection on component 2. 11

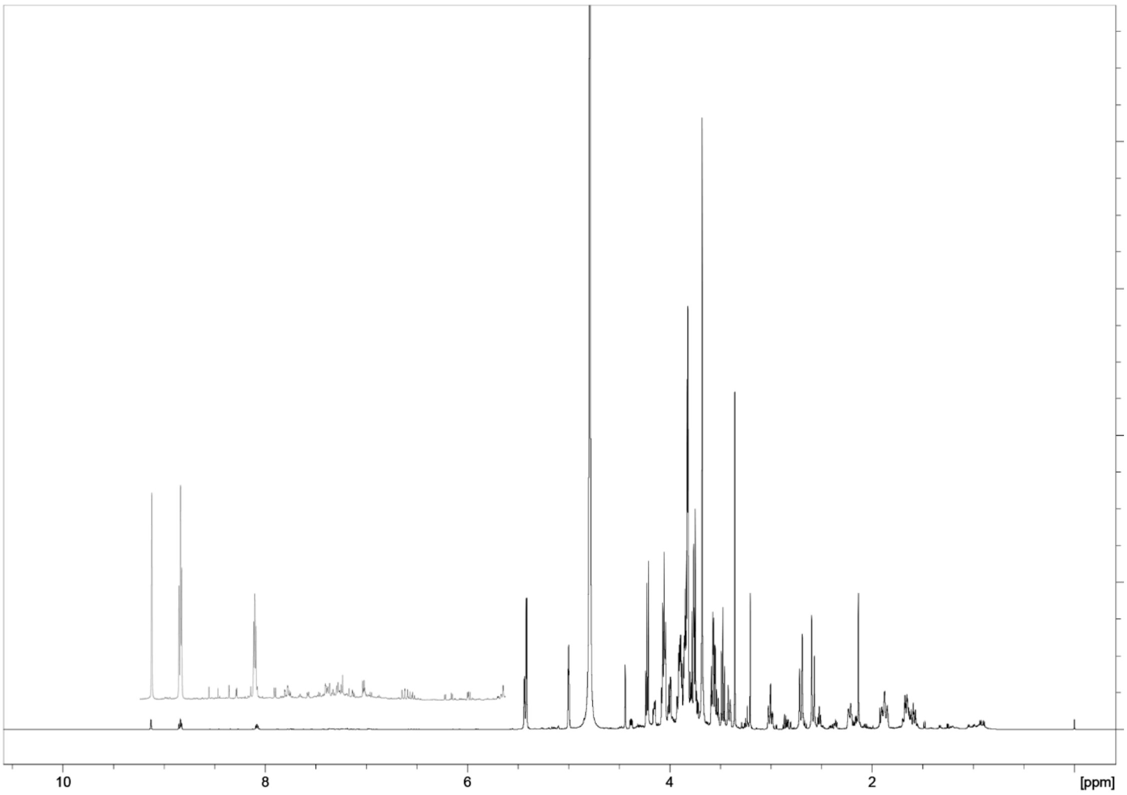

Figure S1. ^{1}H-NMR spectrum in D_2O of polar extract of *Phaseolus* Cannellino (PCANN) at 600 MHz.

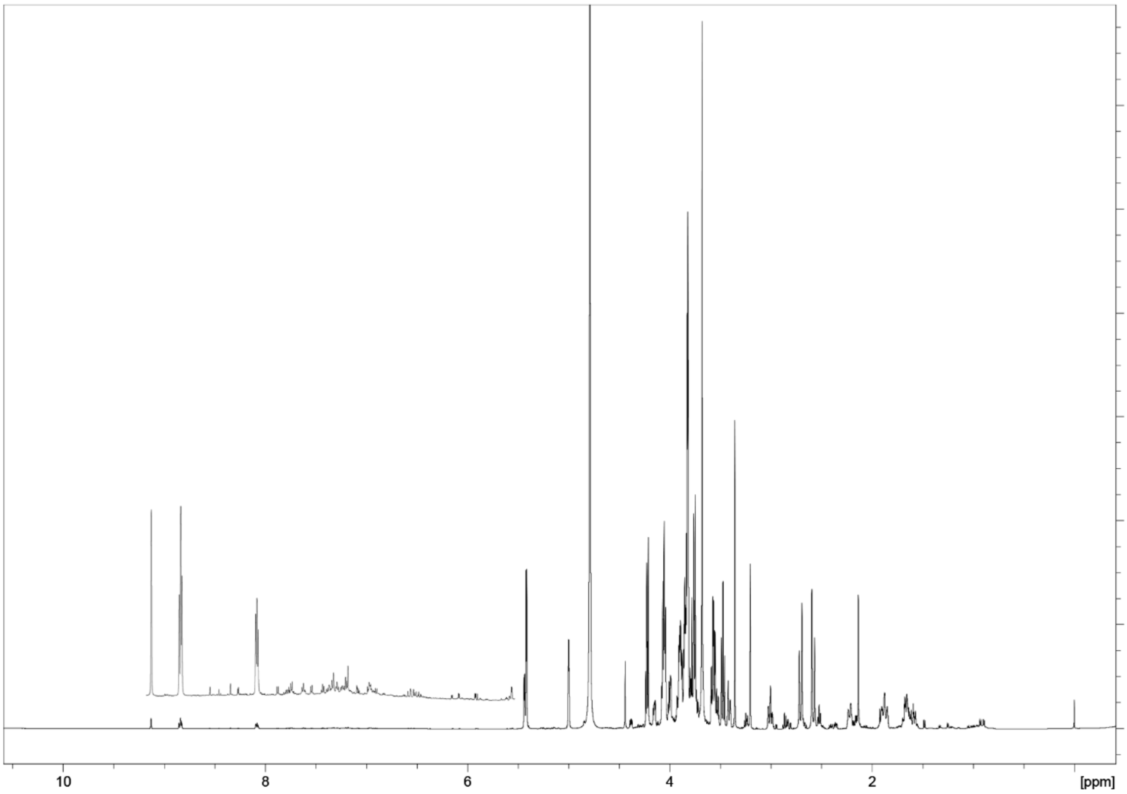

Figure S2. ^{1}H-NMR spectrum in D_2O of polar extract of *Phaseolus* Controne (PCON) at 600 MHz.

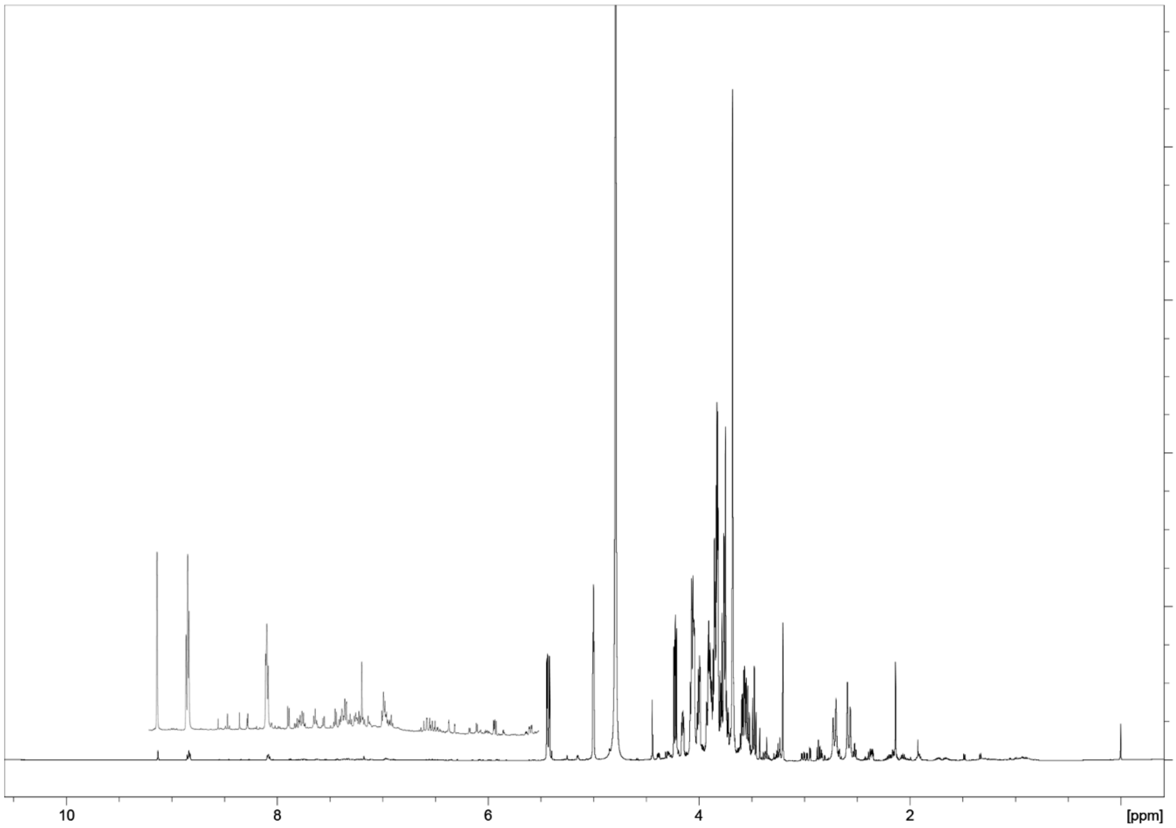

Figure S3. ^{1}H-NMR spectrum in D_2O of polar extract of *Phaseolus* Occhio Nero (PON) at 600 MHz.

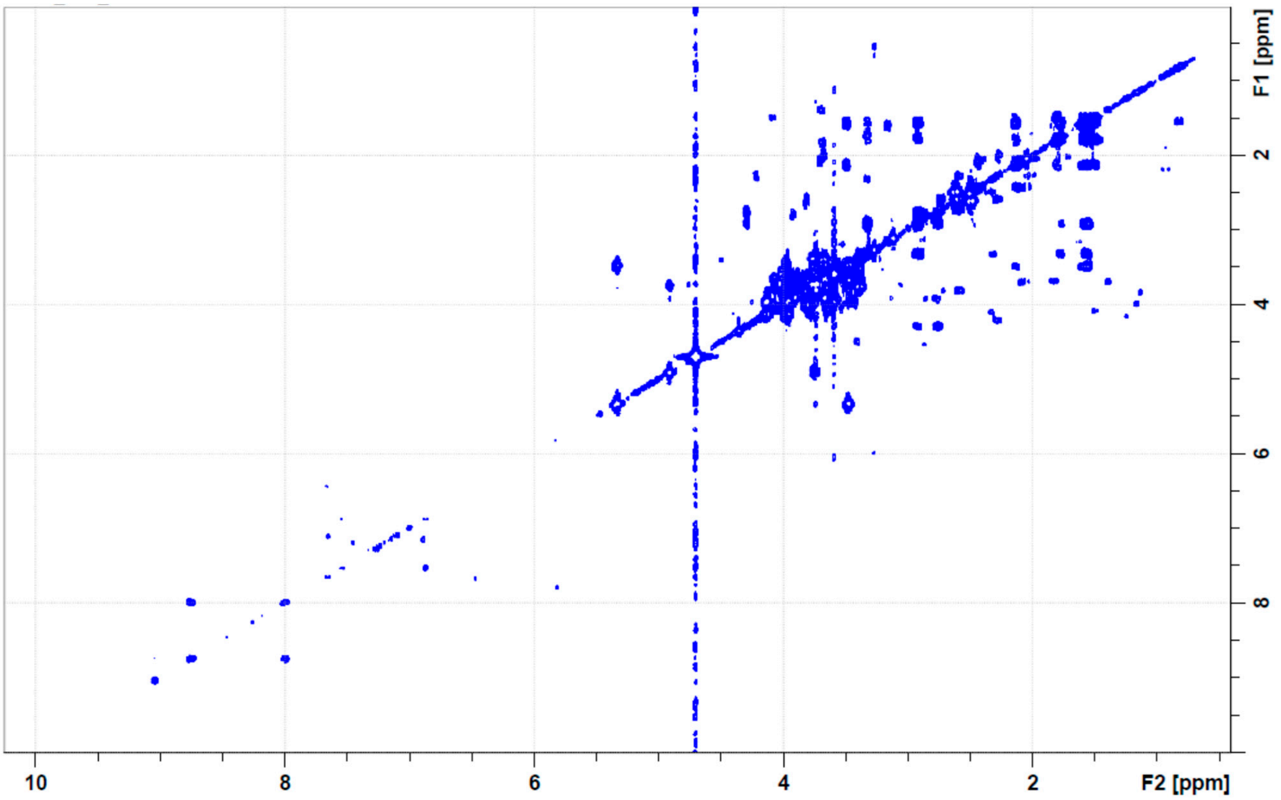

Figure S4. ^{1}H-^{1}H-COSY NMR spectrum in D_2O of polar extract of *Phaseolus* Vellutina (PVEL) at 600 MHz.

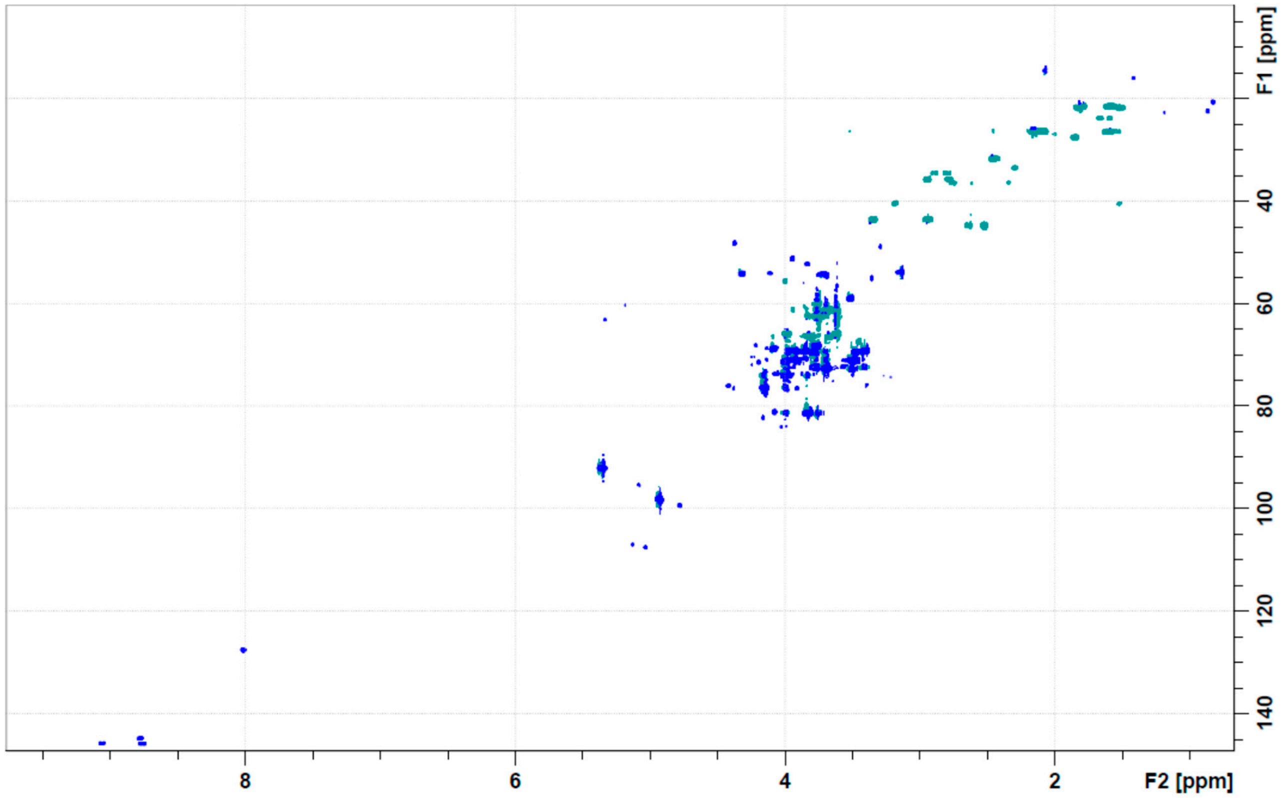

Figure S5. HSQC spectrum in D_2O of polar extract of *Phaseolus* Vellutina (PVEL) at 600 MHz.

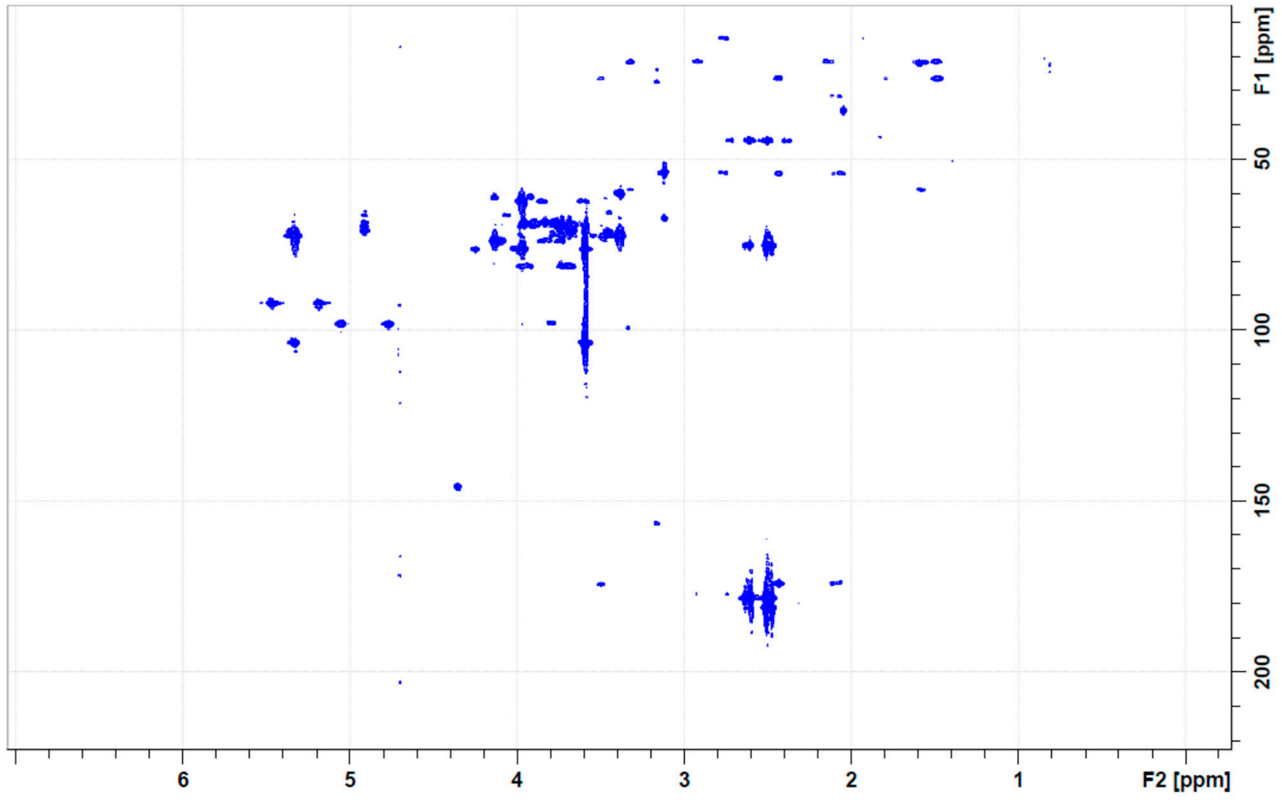

Figure S6. HMBC spectrum in D_2O of polar extract of *Phaseolus* Vellutina (PVEL) at 600 MHz.

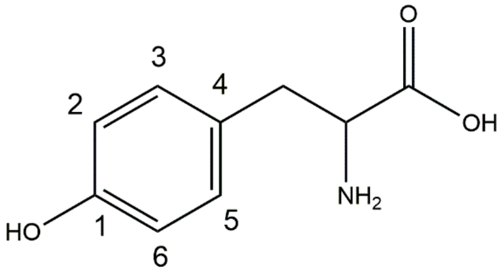

13

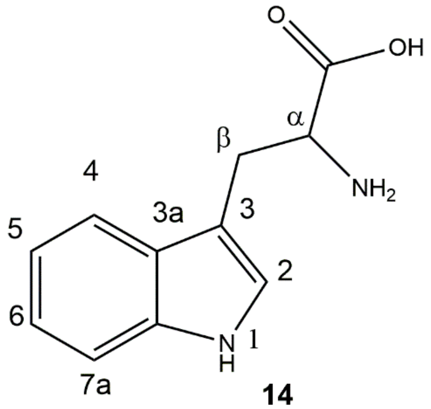

14

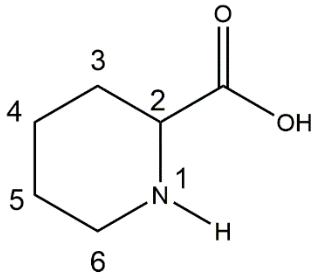

23

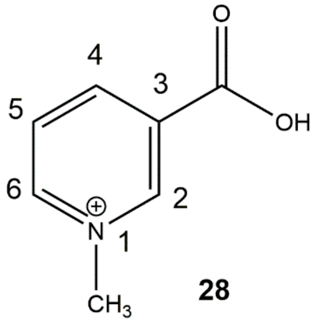

28

Figure S7. Chemical structure of selected metabolites of both polar and apolar extract of *Phaseolus* seeds, namely Tyrosine (**13**, Tyr), Tryptophan (**14**, Trp), Pipecolic acid (**23**, Pip) and Trigonelline (**28**, Tri).

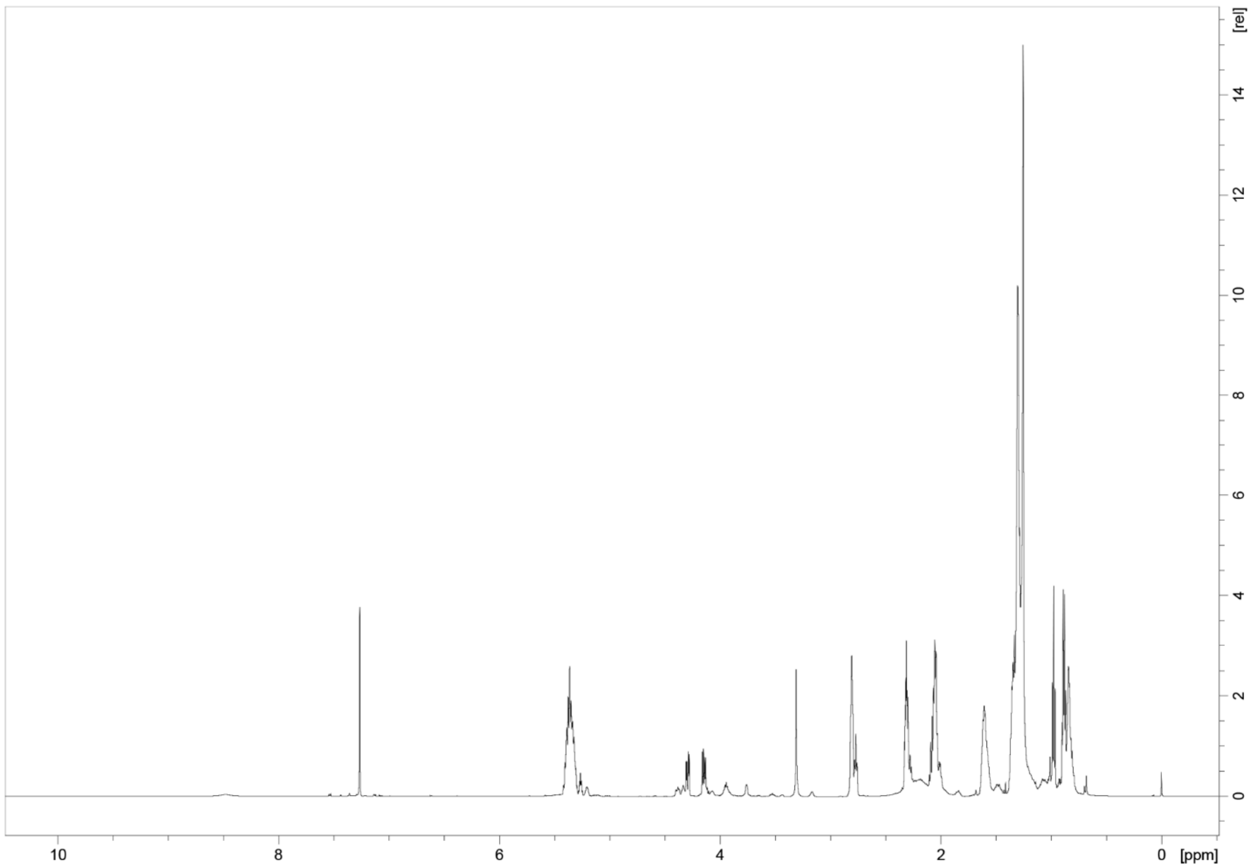

Figure S8. ^{1}H-NMR spectrum in $CDCl_3$ of apolar extract of *Phaseolus* Cannellino (PCANN) at 600 MHz.

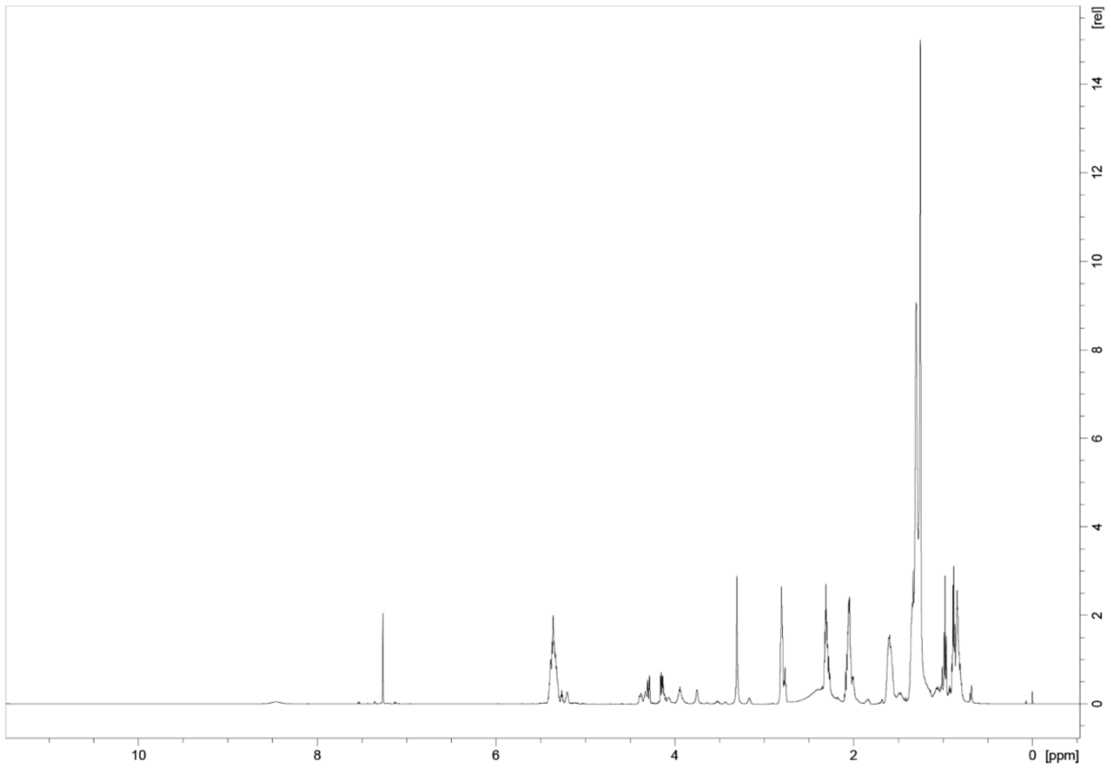

Figure S9. ^{1}H-NMR spectrum in $CDCl_3$ of apolar extract of *Phaseolus* Controne (PCON) at 600 MHz.

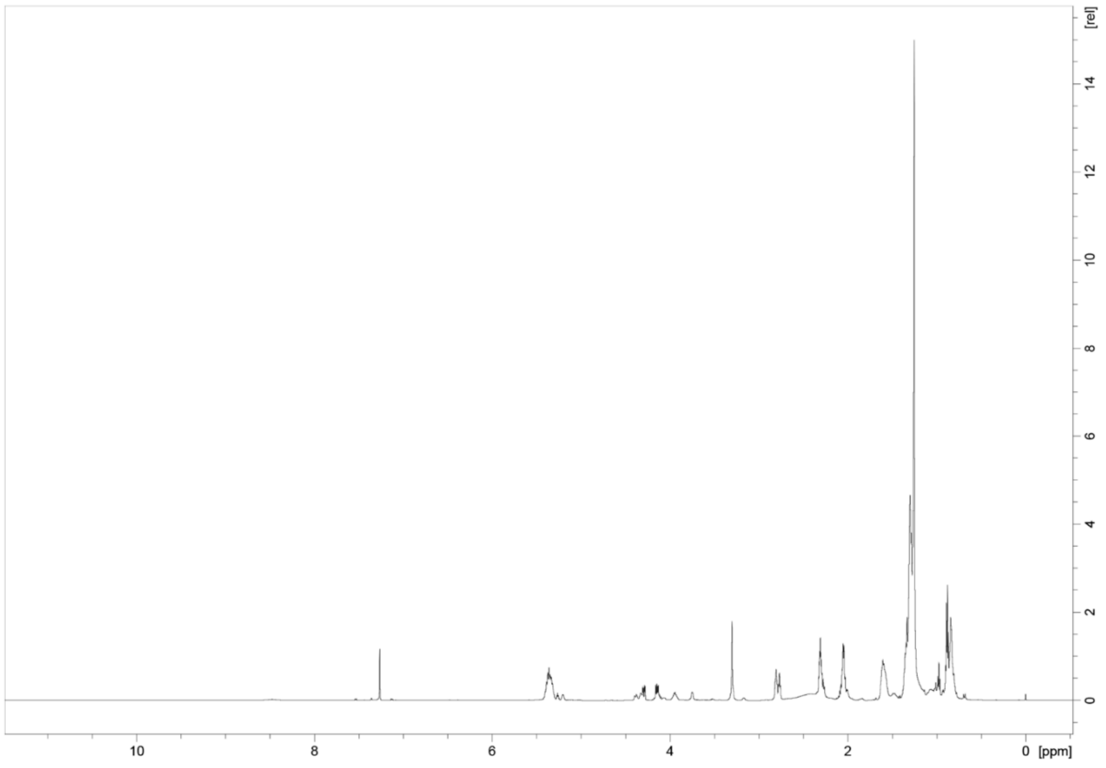

Figure S10. ^{1}H-NMR spectrum in $CDCl_3$ of apolar extract of *Phaseolus* Occhio Nero (PON) at 600 MHz.

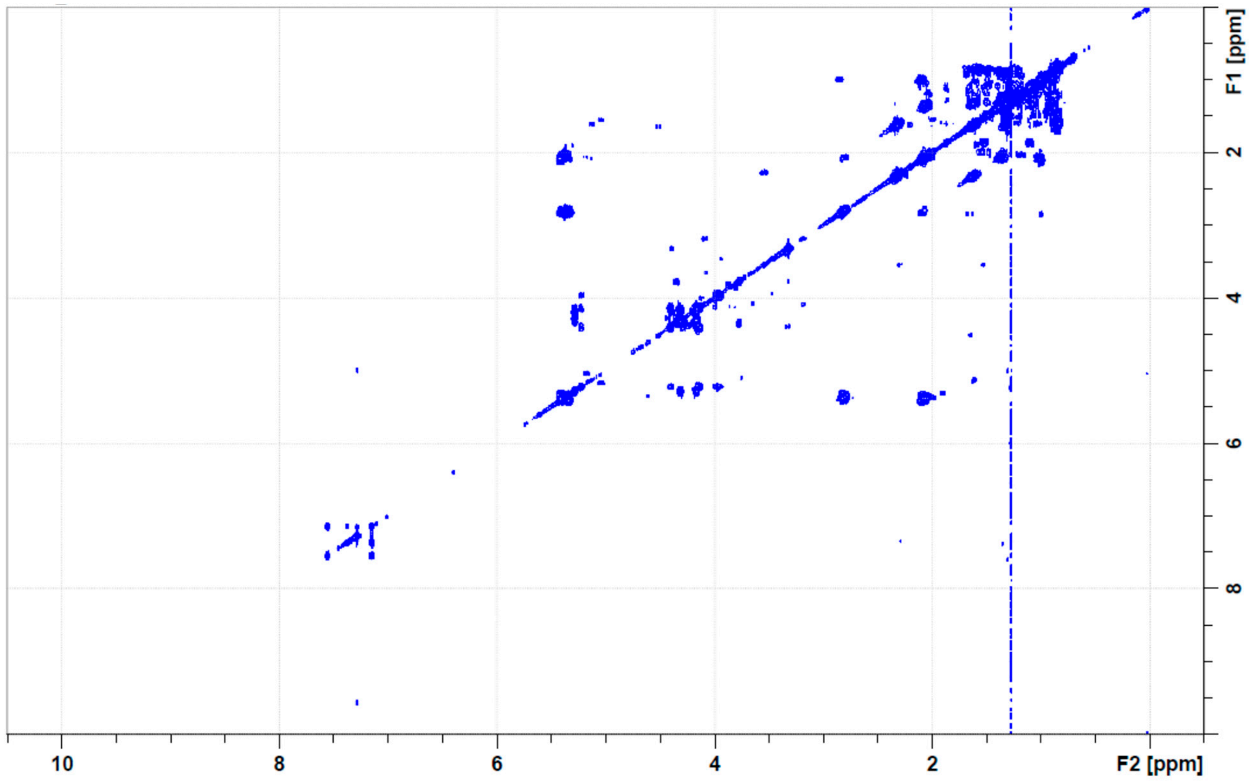

Figure S11. ^{1}H-^{1}H-COSY NMR spectrum in $CDCl_3$ of apolar extract of *Phaseolus* Vellutina (PVEL) at 600 MHz.

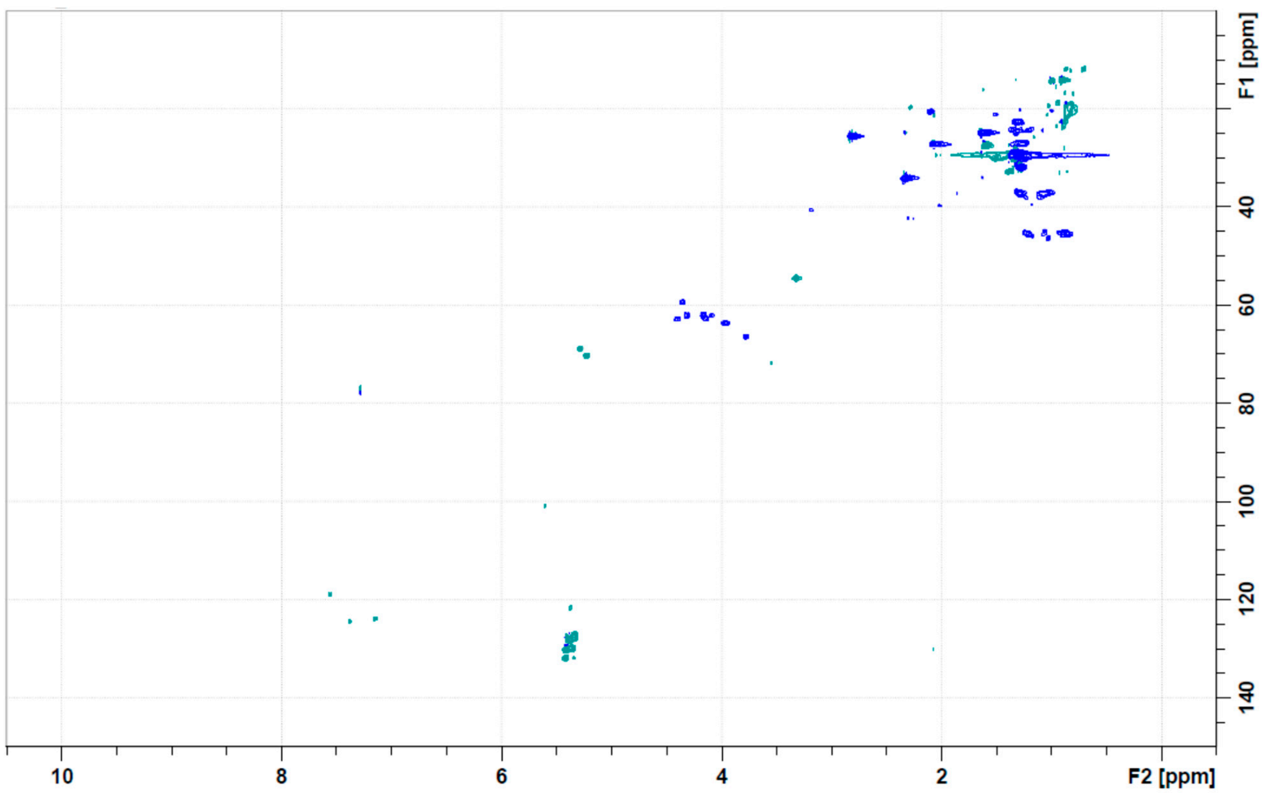

Figure S12. HSQC spectrum in $CDCl_3$ of apolar extract of *Phaseolus* Vellutina (PVEL) at 600 MHz

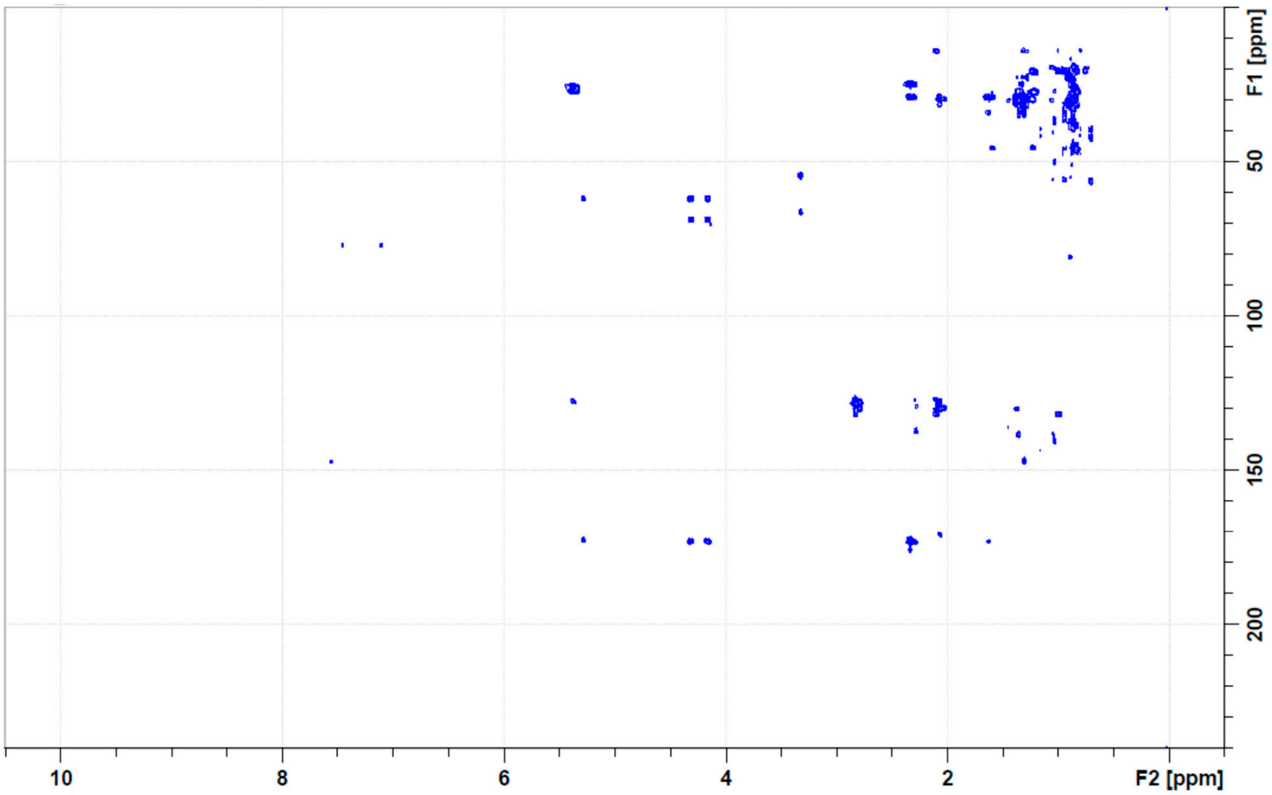

Figure S13. HMBC spectrum in $CDCl_3$ of apolar extract of *Phaseolus* Vellutina (PVEL) at 600 MHz.

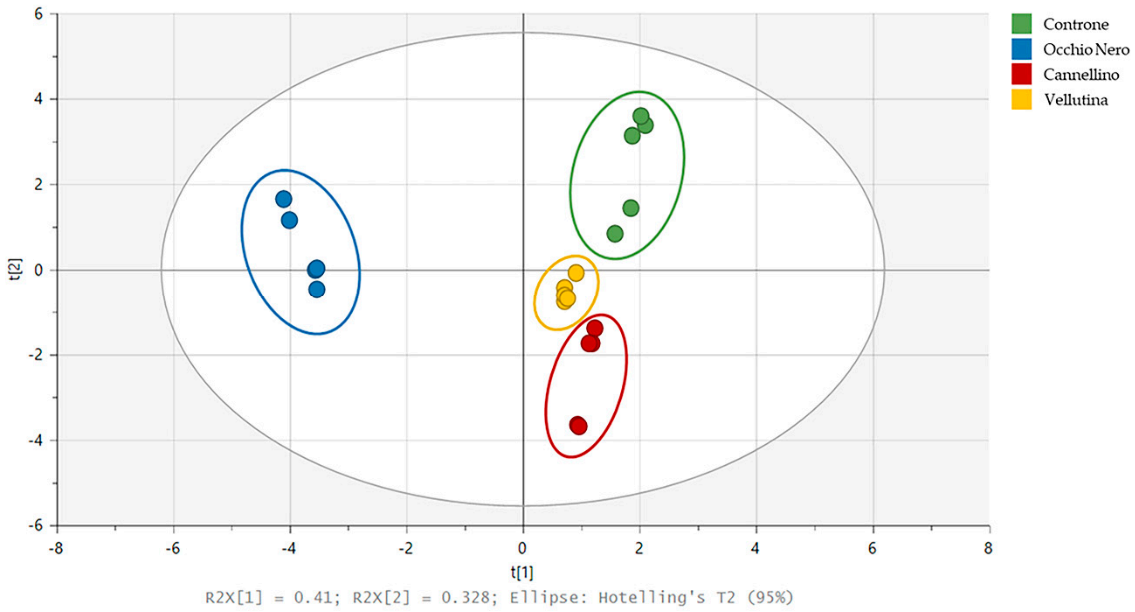

Figure S14. 2D representation of Principal Component Analysis (PCA) of polar extracts of bean varieties. PCANN (red), PCON (green), PVEL (yellow) and PON (blue) are separated in the score plot.

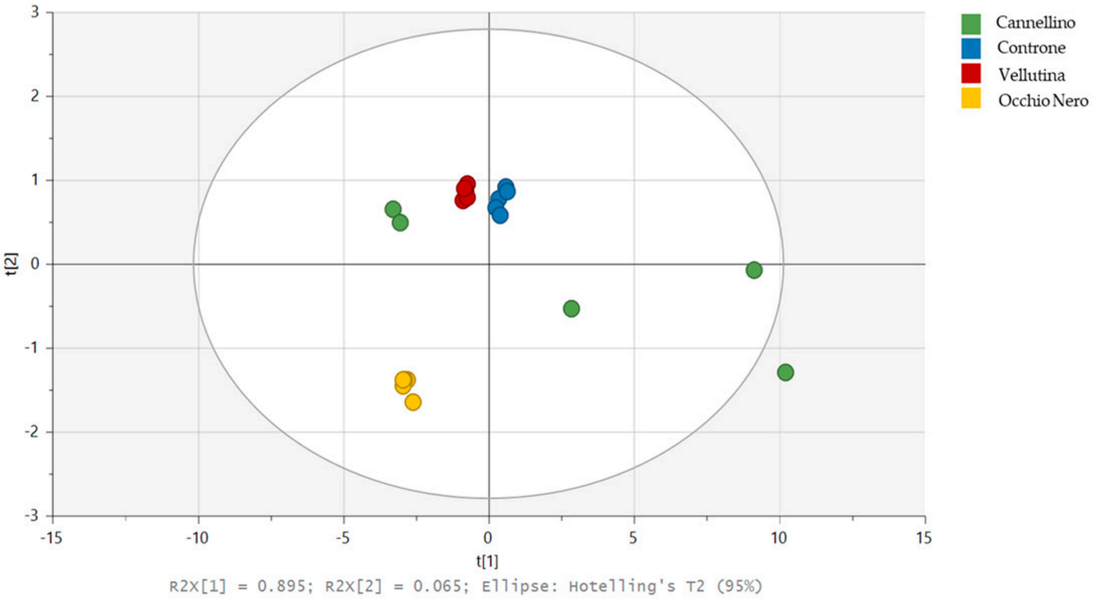

Figure S15. Partial Least Square – Discriminant Analysis (PLS-DA) of the selected metabolites of polar extracts.

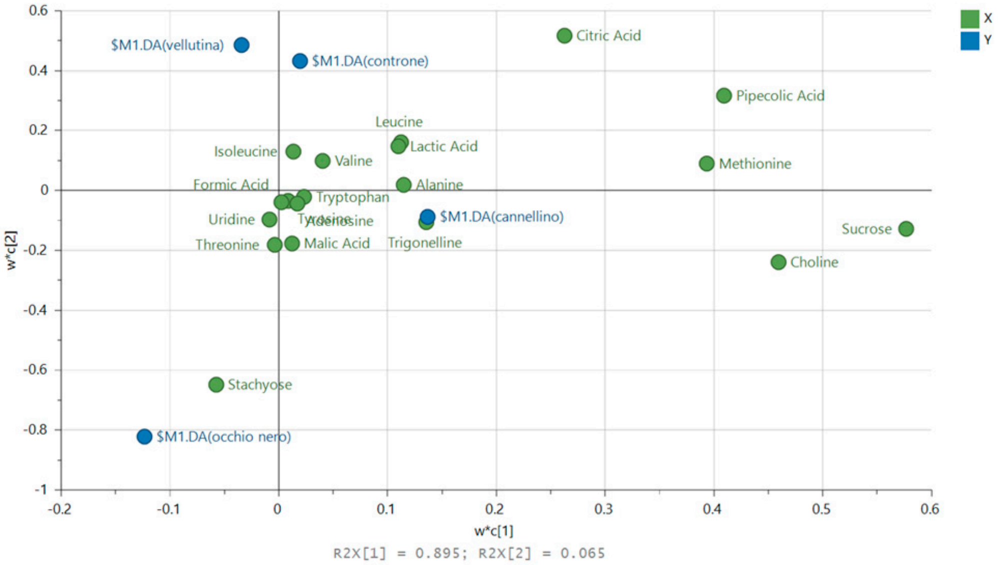

Figure S16. Loading Plot of PLS-DA.

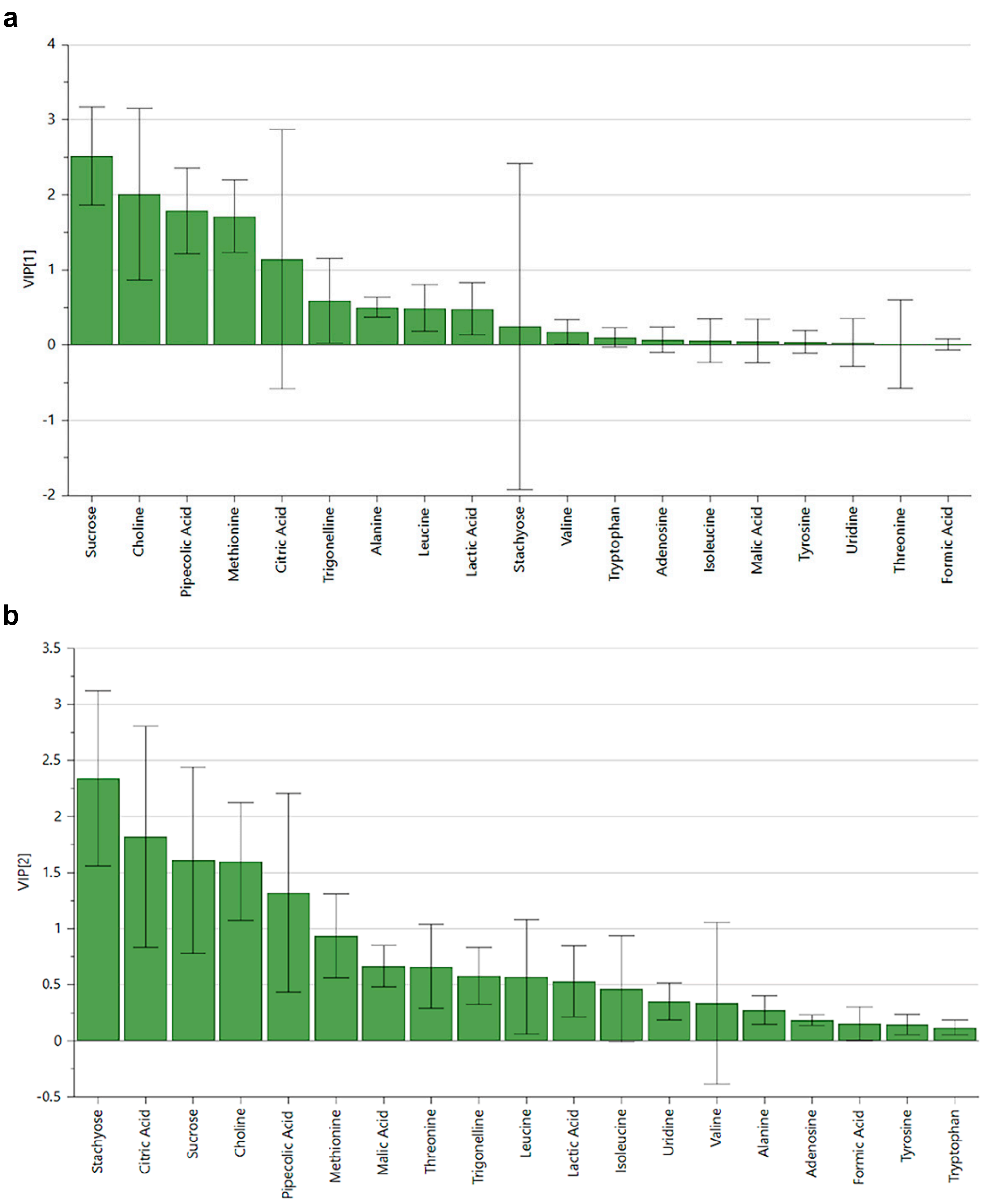

Figure S17. a) Variable Importance in Projection on component 1. b) Variable Importance in Projection on component 2.
